# Hiwi Mediated Tumorigenesis Is Associated with DNA Hypermethylation

**DOI:** 10.1371/journal.pone.0033711

**Published:** 2012-03-16

**Authors:** Sara Siddiqi, Melissa Terry, Igor Matushansky

**Affiliations:** Herbert Irving Comprehensive Cancer Center, Columbia University, New York, New York, United States of America; Vanderbilt University Medical Center, United States of America

## Abstract

Expression of Piwi proteins is confined to early development and stem cells during which they suppress transposon migration via DNA methylation to ensure genomic stability. Piwi's genomic protective function conflicts with reports that its human ortholog, Hiwi, is expressed in numerous cancers and prognosticates shorter survival. However, the role of Hiwi in tumorigenesis has not been examined. Here we demonstrate that (1) over-expressing Hiwi in sarcoma precursors inhibits their differentiation in vitro and generates sarcomas in vivo; (2) transgenic mice expressing Hiwi (mesodermally restricted) develop sarcomas; and (3) inducible down-regulation of Hiwi in human sarcomas inhibits growth and re-establishes differentiation. Our data indicates that Hiwi is directly tumorigenic and Hiwi-expressing cancers may be addicted to Hiwi expression. We further show that Hiwi associated DNA methylation and cyclin-dependent kinase inhibitor (CDKI) silencing is reversible along with Hiwi-induced tumorigenesis, via DNA-methyltransferase inhibitors. Our studies reveal for the first time not only a novel oncogenic role for Hiwi as a driver of tumorigenesis, but also suggest that the use of epigenetic agents may be clinically beneficial for treatment of tumors that express Hiwi. Additionally, our data showing that Hiwi-associated DNA hyper-methylation with subsequent genetic and epigenetic changes favoring a tumorigenic state reconciles the conundrum of how Hiwi may act appropriately to promote genomic integrity during early development (via transposon silencing) and inappropriately in adult tissues with subsequent tumorigenesis.

## Introduction

In all model systems examined, Piwi family members are expressed in stem cells, including germ and hematopoietic, and are essential for germ line and/or somatic stem cell self-renewal [1], [2], [3], [4]. Although the exact mechanism is still unclear even in the most studied models (e.g., drosophila, mice [1], [2], [3], [4]), Piwi appears to ensure stem cell maintenance by inhibiting transposon migration [5], [6] during early development via an indirect (since Hiwi has no known direct chromatin modifiying function) upregulation of epigenetically based silencing machinery (i.e., DNA methylation) [7], [8], [9]. Specifically, previous studies have shown that transposon-specific DNA-methylation was reduced and transposon activity was elevated following silencing of Hiwi (or its orthologs). Although transposons promote evolutionary diversity in lower organisms, their unchecked migration in higher organisms can result in disruption of genomic integrity [10] and thus Piwi proteins may have developed as an evolutionary defense system for multi-cellular species.

Based on the data that implicate Piwi in transposon silencing, maintenance of genome integrity and exclusively embryonic and/or stem cells expression, it is surprising that a growing body of studies reveal that Hiwi, the human ortholog of Piwi, is expressed in a diverse group of cancers including: seminomas [11], pancreatic [12] and gastric [13] adenocarcinomas, squamous cell carcinomas [14]; and sarcomas [15]. In sarcomas [15] and pancreatic [12] cancers higher Hiwi mRNA levels were predictive of worse clinical outcomes. These data lead to an obvious conundrum: why would a gene that is critical for maintaining genome integrity during development be highly expressed in cancer? Since the above studies focused exclusively on Hiwi expression levels in cancer cells, mechanistic insight into Hiwi's role in tumorigenesis remains completely unexamined.

Herein we explore the necessity and sufficiency of Hiwi for tumorigenesis and maintenance of the tumorigenic phenotype using mesenchymal stem cells and sarcomas in both in vitro and transgenic models. Surprisingly we find that Hiwi is directly tumorigenic. We go on to show that Hiwi mediated DNA methylation is associated with tumor suppressor gene silencing, thus potentially accounting for Hiwi-mediated tumorigenesis. Our data reconcile the conundrum of how Hiwi may act appropriately to promote genomic integrity during early development (via transposon silencing) and inappropriately in adult tissues with subsequent tumorigenesis.

## Results

### Hiwi inhibits differentiation and promotes sarcomagenesis

**Figure 1:**
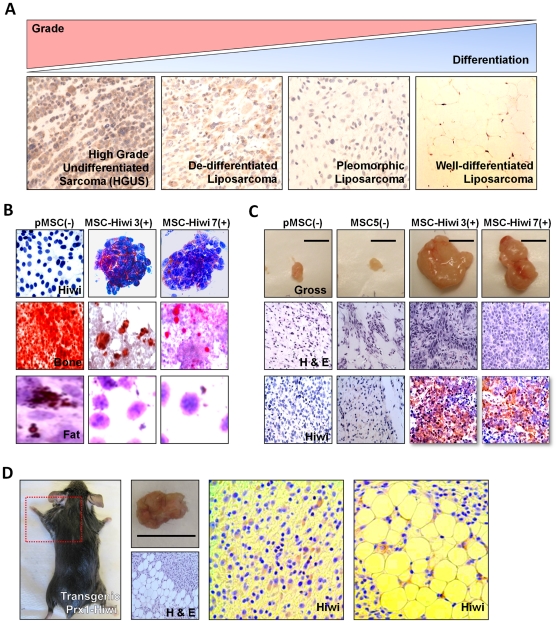
Hiwi inhibits differentiation and promotes sarcomagenesis. (A) Immunohistochemical (IHC) analysis of Hiwi on a human sarcoma tissue microarray (TMA). Ten cases of each subtype (present in triplicate) were scored from 0 to 2 blindly by sarcoma pathologists. Representative pictures are shown. (B) Top row: IHC analysis reveals Hiwi-MSC clones (3 and 7) have a distinct clumped morphology and highly express Hiwi. Middle row: Day 21 in osteogenic media. Calcium matrix formation measured by Alizarin Red S stain is decreased in Hiwi-MSCs, approximately 100% versus 5% of cells staining. Bottom row: Day 21 in adipogenic media. Lipid formation measured by Oil Red O stain is decreased in Hiwi-MSCs, approximately 25% versus 1% of cells staining. Experiments were performed in triplicate and representative pictures are shown. (C) Top row: Xenograft tumors derived from Hiwi-MSCs. Middle row: H&E analysis reveals tumors from Hiwi-MSCs are undifferentiated sarcomas. Bottom row: By IHC analysis, tumors from Hiwi-MSCs continue to express Hiwi. All experiments were performed in triplicate. (D) Transgenic mouse model of Hiwi forms sarcomas (left panel) with both well-differentiated and poorly-differentiated sections (H&E panel) and continues to express Hiwi (right panels).

Following previous PCR-based observations that Hiwi is expressed in sarcomas [15] and that its expression correlates with prognosis [15], we examined Hiwi protein levels via immunohistochemistry (IHC) in a large primary human sarcoma tissue microarray (TMA) composed of numerous soft-tissue sarcomas (previously described by us [18]). Ten cases of each sarcoma subtype (present in triplicate) were scored from 0 to 2 blindly by sarcoma pathologists. To examine the relationship between cellular differentiation and tumor grade, we focused on a panel of liposarcomas, since we have previously shown that high grade undifferentiated sarcomas (HGUS), dedifferentiated liposarcomas, pleomorphic liposarcomas, and well differentiated lipoarcomas correspond to a gradual adipocytic differentiation spectrum [23]). We noted that Hiwi levels correlated directly with grade and indirectly with tumor cellular differentiation. Similar observations were made for other sarcoma subtypes present on the TMA as well (e.g., leiomyosarcomas (data not shown)) (**Figure 1A**). Overall Hiwi is expressed at significantly higher levels (p<0.005) in undifferentiated sarcoma subtypes compared with more well-differentiated subtypes (**Figure S1**). Both tumor grade and tumor cellular differentiation have been shown to correlate with clinical prognosis for sarcomas [24]. These data suggest that Hiwi is associated with the undifferentiated mesenchymal tumorigenic state and thus by definition designates a poor prognostic outcome [24].

To assess whether Hiwi merely associates with inhibition of differentiation and tumorigenesis, or whether Hiwi may directly inhibit differentiation and promote sarcomagenesis, we expressed Hiwi in sarcoma precursors (i.e., mesenchymal stem cells, MSCs; **Figure 1B** **; Figure S2 and Supporting Information S1**). Hiwi-expressing MSCs (Hiwi-MSC-3&7) and parental MSCs were cultured in either adipogenic or osteogenic differentiation medium [18] and, as per standards of the field, were assayed for phenotypic maturation with either Alizarin-Red-S for bone/calcium mineralization or with Oil-Red-O for fat accumulation. Hiwi-expressing MSCs (Hiwi-MSC-3&7; columns 2 and 3, **Figure 1B**) show impaired differentiation into both osteogenic (second row, **Figure 1B**) and adipogenic (third row, **Figure 1B**) mesenchymal lineages as compared to parental MSCs (pMSC; column 1, **Figure 1B**), which readily accumulate mineralized calcium and form adipocytic foci. We then inoculated Hiwi-expressing MSCs (Hiwi-MSC-3&7), parental MSCs and a non-expressing, antibiotic resistant MSC clone (MSC5; isolated from our initial transduction) into NOD-SCID-Gamma mice. Tumors formed from Hiwi-MSC -3&7 inoculations after five weeks. Morphological analysis (Fabrizio Remotti MD, Department of Pathology, CUMC) showed that Hiwi- MSC 3&7-derived tumors were high grade sarcomas. Further IHC analysis also showed that they expressed Hiwi. In contrast to Hiwi-MSC 3&7, parental MSCs and MSC5 formed small fibrous plaques devoid of tumor cells (**Figure 1B, C** **; Figure S3 and Supporting Information S1**).

We further generated transgenic mice expressing Hiwi under the control of the early mesodermally restricted Prx1 enhancer element [21]. 51 Prx1-Hiwi expressing progeny have been generated to date. Early sarcoma formation was identified in two (4%) of 51 Prx1-Hiwi mice at 12 weeks, in a known Prx1 distribution, that contain both well-differentiated and high grade components and express Hiwi (**Figure 1D**). No other developmental or pathological abnormalities were observed in these Prx-Hiwi transgenic mice. No sarcoma formation was found in wildtype, Hiwi-non-expressing littermate control mice. Although we realize this is a low tumor incidence, it is still significantly higher than deletion of the retinoblastoma tumor suppressor under the same promoter [25]; suggesting to us that the low incidence rate may under-represent Hiwi's tumorigenic potential in this model. Additionally, soft-agar oncogene colony formation assays show that Hiwi is sufficient to transform MSCs (p<0.05) and further cooperates with Ras (**Figure S4 and Supporting Information S1**; p<0.005). Taken together our data suggest that Hiwi functions as an oncogene.

### Hiwi expression correlates with DNA methylation

**Figure 2:**
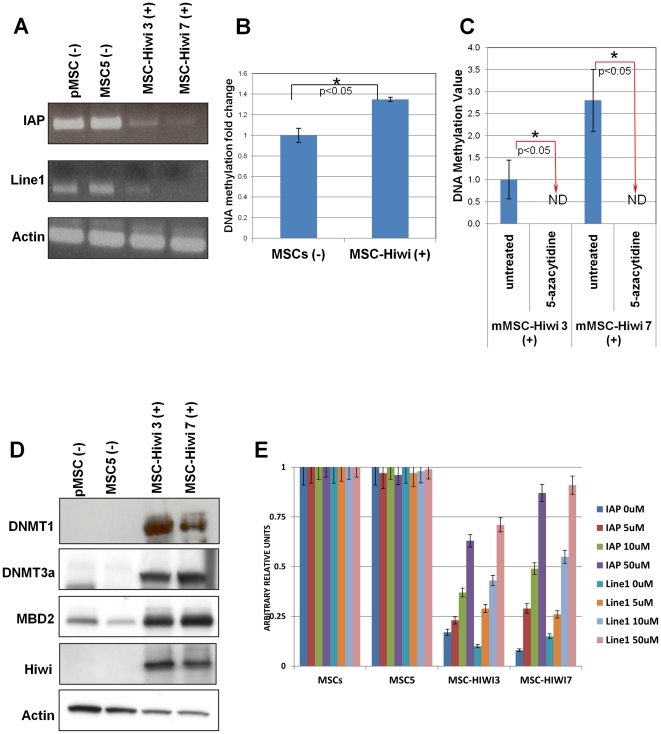
Hiwi expression correlates with DNA methylation. (A) IAP and Line1 transposon expression is decreased in Hiwi-MSCs. IAP and Line1 semi-quantitative PCRs were run for 25 cycles. Actin is a loading control. (B) Global DNA methylation is increased in Hiwi-MSCs. Error bars represent standard error. * = p<0.05 by Student's T Test (C) Global DNA methylation is decreased to non-detectable levels (ND) after 18 h treatment with 10 uM 5-azacytidine in Hiwi-MSCs. Lower doses of 5-azacytidine (including 1 uM) did not change DNA methylation levels. Error bars represent standard error * = p<0.05 by Student's T Test (D) Western blots of indicated proteins reveal increased expression of DNMT1, DNMT3a and MBP2 in Hiwi-MSCs. Actin is a loading control. (E) Treatment of Hiwi expressing MSCs with 0 uM, 5 uM, 10 uM, or 50 uM 5-azacytidine for 18 h restores IAP and Line1 transposon expression in a dose-dependent manner. IAP and Line1 quantitative RT-PCRs were run and parental MSC of each 5-azacytidine concentration were used to normalize the rest of the samples at that concentration. Error bars represent standard error. All experiments were performed in triplicate.

Since Hiwi orthologs have been implicated in transposon silencing via DNA methylation [5], [6], we examined both the expression levels of two common transposons (IAP and Line1) and also global DNA-methylation levels in Hiwi-MSCs (Hiwi-expressing MSCs, clones 3 and 7). Global DNA methylation was assessed by an ELISA-like assay, using an antibody against 5-Methyl Cytosine, which detects cytosine methylation at both CpG sites and non-CpG sites. Both IAP and Line1 levels were severely reduced (**Figure 2A**) while global DNA methylation was significantly increased (approximately 40%, p<0.05; **Figure 2B**) in Hiwi-MSCs as compared to non-Hiwi-expressing MSCs (pMSCs & MSC5); suggesting that Hiwi expression leads to increase in DNA methylation. We then checked whether the Hiwi-associated increase in global DNA methylation is reversible with the DNA-methyltransferase inhibitor, 5-azacytidine (Vidaza). Indeed, 5-azacytidine treatment of Hiwi-MSCs completely (100%, p<0.05) reverses the increase in global DNA-methylation associated with Hiwi (**Figure 2C**) suggesting that DNMTs may be crucial intermediaries in Hiwi-mediated methylation. Of note, we did not observe any decrease in global DNA methylation following treatment of non-Hiwi-expressing MSCs with 5-azacytidine (data not shown), which may be reflective of the lower baseline global DNA methylation levels in those cells.

Having established a direct correlation between Hiwi, DNA methylation, and Hiwi-associated DNA methylation reversibility via DNA-methyltransferase inhibitors, we examined the levels of proteins known to modulate DNA methylation: DNA methyltransferase 1 (DNMT1), DNA methyltransferase 3 (DNMT3a), and methyl-binding protein 2 (MBD2). All three were found to increase in the presence of Hiwi (**Figure 2D**). Interestingly, we find no expression changes of DNMT3b in Hiwi-MSCs (data not shown). Although the exact mechanism of how Hiwi promotes an upregulation of DNMTs remains unclear, both here and in all other developmental models where these proteins have been much more thoroughly explored, the exact mechanism of Piwi is unknown [1], [2], [3], [4], [5], [6], [7], [8], [9]. Finally, we show that treatment of Hiwi-MSCs with 5-azacytidine can reverse Hiwi-mediated transposon silencing (**Figure 2E** **; Figure S5**), restoring IAP and Line1 transposon expression back to levels comparable with parental MSCs as the concentration of 5-azacytidine increases. No such changes in transposons were observed in non-Hiwi expressing cells, pMSCs and MSC5 (**Figure 2E** **,**). Taken together, these data suggest that Hiwi controls transposon expression directly and is associated with DNA methylation in Hiwi-transformed MSCs.

### Downregulation of Hiwi decreases DNA-methylation and limits tumorigenic growth

**Figure 3:**
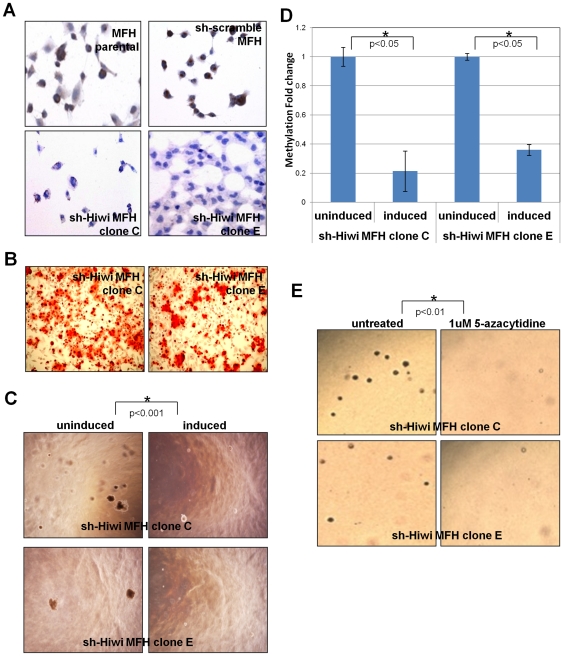
Down-regulation of Hiwi decreases DNA-methylation and limits tumorigenic growth. (A) Verification of inducible sh-Hiwi MFH clones by immunohistochemistry. sh-Hiwi MFH clones C and E were induced with doxycycline for 3 days before IHC analysis. (B) Day 21 in osteogenic media. Calcium matrix formation measured by Alizarin Red S stain is restored in sh-Hiwi MFH clones C and E (approximately 50% of cells staining), induced with doxycycline 7 days before addition of differentiation media and continued in doxycycline-spiked media during differentiation. (C) Doxycycline-induced sh-Hiwi MFH cells at 4 weeks in colony forming assay show decreased colony formation, as compared to uninduced sh-Hiwi MFH cells. p<0.001 for clone C and p<0.005 for clone E by Student's T Test (D) Global DNA methylation is decreased in induced sh-Hiwi MFH cells. Cells were induced with doxycycline for 3 days before DNA was collected and assayed. Error bars represent standard error. * = p<0.05 by Student's T Test (E) Untreated sh-Hiwi MFH cells or treated with 1 uM 5-azacytidine at 4 weeks in colony forming assay. 5-azacytidine treatment decreases colony formation capacity. Combined p<0.01 by Student's T Test. All experiments were performed in triplicate. Representative pictures are shown.

Having shown that Hiwi expression in sarcoma precursors leads to sarcomagenesis, we queried if Hiwi is necessary for maintenance of the sarcoma phenotype. We first identified an undifferentiated sarcoma cell line, MFH (previously characterized by us [17], [18], endogenously expressing Hiwi (**Figure S6 and Supporting Information S1**). We then engineered MFH cells to express doxycycline-inducible Hiwi-short hairpin; (dox-ind-sh-Hiwi MFH; **Figure 3A**, top left panel). Both parental MFH cells and sh-scramble MFH negative control cells continue to express high levels of Hiwi. In contrast, doxycycline-induced sh-Hiwi MFH cells have dramatically reduced Hiwi levels (**Figure 3A**; Clones C and E were chosen for further analyses). Although MFH cells do not undergo induced mesenchymal differentiation [17], [18], dox-ind-sh-Hiwi MFH cells are able to undergo mesenchymal differentiation following doxycycline induction and exposure to differentiation medium (**Figure 3B**, osteogenic differentiation is shown with Alizarin Red S). In contrast, in the absence of doxycycline (parental MFH cells) rapidly overgrow and die (data not shown and as previously described [18], [26]). Colony formation is significantly reduced to about 40% of untreated control for dox-ind-sh-Hiwi MFH clone C and to about 20% of untreated control for dox-ind-sh-Hiwi MFH clone E (p<0.001 for clone C and p<0.005 for clone E; **Figure 3C**). Concurrent with down-regulation of Hiwi, we observe a significant decrease in global DNA-methylation (approximately 70%, p<0.05; **Figure 3D**). In agreement, 5-azacytidine treatment of sh-Hiwi MFH cells (in the absence of doxycycline) results in elimination of colony formation (**Figure 3E**), similar to that seen in doxycycline-induced sh-Hiwi MFH (**Figure 3D**). Colony formation during 5-azacytidine treatment was reduced to about 25% of untreated control for sh-Hiwi MFH clone C and to about 50% of untreated control for sh-Hiwi MFH clone E (combined p<0.01 for both clones). Of note, we previously published on the general insensitivity of sarcoma cell lines to 5-azacytidine treatment [26], but now suggest that Hiwi expressing tumors may be an exception. Taken together these results indicate that Hiwi is necessary for maintenance of the tumorigenic phenotype of Hiwi-expressing cells.

**Figure 4:**
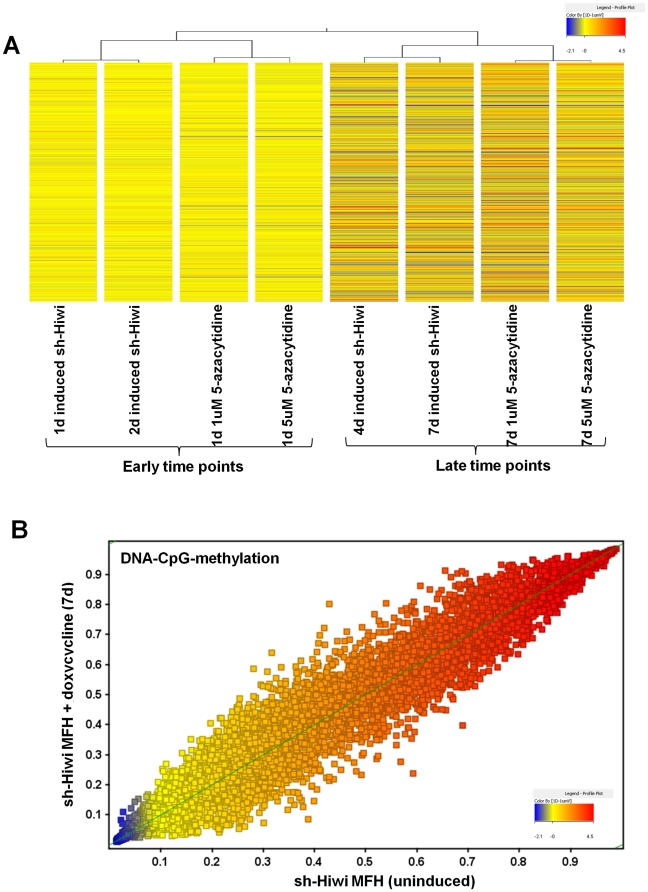
Hiwi down-regulation and 5-azacytidine treatment are mechanistically similar. (A) Gene expression profiles of sh-Hiwi MFH cells treated with 1 uM or 5 uM 5-azacytidine cluster with gene expression profiles of doxycycline-induced sh-Hiwi MFH cells, using the whole gene set from both treatments. (B) Meth27 Illumina array of methylation of 27000 CpG site changes in sh-Hiwi MFH cells uninduced (x-axis) or induced with doxycycline for 7 days (y-axis). Graph shows all CpG islands and reveals no global shift in CpG methylation.

Global DNA methylation decrease and growth delay were common to both Hiwi down-regulation and DNA-methyltransferase inhibitor treatment, but to address whether the specific mechanism was the same, we performed a temporal gene expression profiling (Affymetric U133 Plus 2.0 Arrays) on sh-Hiwi MFH cells after Hiwi down-regulation (via doxycycline induction) or 5-azacytidine treatment. Using unsupervised hierarchical clustering of the whole gene sets of both conditions, we find that early time points (24–48 hrs) of Hiwi down-regulation associate with early time points (24–48 hrs) of 5-azacytidine treatment, and similarly, longer down-regulation (4–7 d) of Hiwi associates with longer treatment with 5-azacytidine (**Figure 4A**). Overlap of differentially expressed genes in both array sets (**Figure S7 and Supporting Information S1**) shows that at early time points, 75% of the genes that are differentially expressed following Hiwi down-regulation are also differentially expressed during 5-azacytidine treatment; and over 99% of these overlapping gene changes trend similarly. At later time points, 50% of the genes that are differentially expressed following Hiwi down-regulation are also differentially expressed following 5-azacytidine treatment, with 93% of these overlapping gene changes trending similarly. These data suggest that 5-azacytidine treatment of MFH cells mechanistically mimics Hiwi down-regulation by targeting the same genes.

We then examined the methylation changes in 27,000 CpG island covering 14,495 genes that occur during Hiwi down-regulation in doxycycline-inducible sh-Hiwi MFH cells using Illumina Meth27 arrays before and after 2, 4 and 7 days of doxycycline induction. Despite our data showing that global DNA methylation directly correlates with Hiwi expression (**Figures 2** **and** **3**), no overall change in CpG island methylation was observed following Hiwi down-regulation (**Figure 4B**). We did identify 18 CpG islands that decreased by at least 10% (i.e., beta>0.1) (**Figure S8 and Supporting Information S1; Table S1**) following Hiwi down-regulation after 7 days in both of our inducible sh-Hiwi MFH clones C & E (no significant changes in DNA methylation were observed at earlier time points). However, the 17 genes associated with these 18 CpG islands did not show a simultaneous increase in gene expression following Hiwi down-regulation nor did they show a decrease in Hiwi-MSCs as compared to parental MSCs (**Figure S9 and Supporting Information S1**). And finally an equal number of CpG islands can readily be observed (**Figure 4B**) that gain CpG methylation following Hiwi down-regulation. These data suggest that CpG methylation status of genes (at least the 14,495 on the Illumina Meth27 array) are not affected; and thus that the overall change in global DNA methylation observed may be accounted by either DNA-methylation of repetitive elements (non-gene regions) or non-CpG gene methylation.

### Assessment of Hiwi target genes

**Figure 5:**
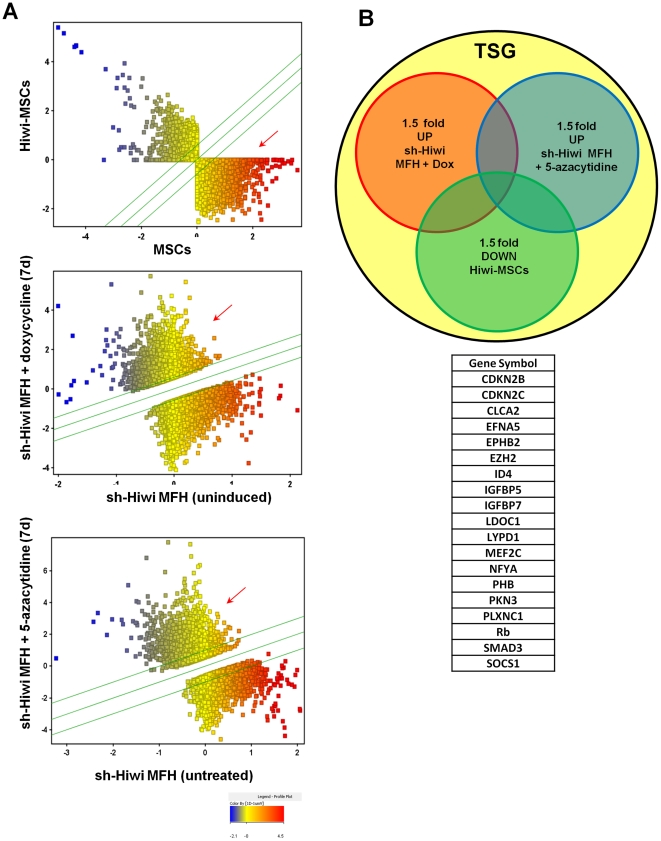
Assessment of Hiwi target genes. (A) Top panel: Affymetrix 430 2.0 array of gene expression changes in parental MSCs (x-axis) or Hiwi-MSCs (y-axis). Affymetrix U433 array of gene expression changes in sh-Hiwi MFH cells uninduced (x-axis) or induced for 7 days with doxycycline (y-axis) (middle panel) or untreated (x-axis) and after 7 days of 1 uM 5-azacytidine (y-axis) (bottom panel). Arrow indicates genes used in overlap analysis. (B) Overlap of Tumor Suppressor Genes (TSG) as described. All 19 overlapping TSGs are listed here.

To explore further the potential relationship between Hiwi associated DNA methylation and the resultant effects on genes we performed global gene expression analysis and subsequently focused on a group of tumor suppressor genes (TSGs) [27] that were at least 1.5 fold: (1) down-regulated in Hiwi-MSCs as compared to parental MSCs; (2) up-regulated in dox-ind-sh-Hiwi MFH cells following 7 days of doxycyline; and (3) to ensure that these genes were DNA methylation dependent, further up-regulated following 5-azacytidine treatment of MFH cells (**Figure 5A**). This overlap gave rise to 19 genes (**Figure 5B**). Since Rb1 was identified in this set and its methylation has been thoroughly studied, we further assayed its promoter via bisulfite sequencing as well as the Line1 regulatory element (**Figure S10**). In agreement with our CpG promoter methyl array results, there are no changes in the methylation status of the Rb1 promoter CpG sites as Hiwi levels change (**Figure S10**, top and middle graphs). Additionally, we find that the Line1 transposon CpG sites also remain unchanged as Hiwi level change (**Figure S10**, bottom graph). These data suggest that because no methylation changes occur in these methylation-dependent genes, Hiwi-associated DNA methylation is non-CpG sites methylation. We went on to further examine the methylation of Line1 and IAP transposon regions before and after treatment with 5-azacytidine in Hiwi-MSCs (**Figure S11 and Supporting Information S1**). The methylation of these regions remains unchanged during 5-azacytidine treatment, further suggesting that Hiwi-associated methylation is non-CpG methylation. In agreement with our promoter CpG methyl array (**Figure 4B**), no methylation changes at any CpG islands (increase or decrease) in these DNA methylation dependent genes (**Figures 2** **,** **3** **and** **5A**) were observed (**Figure S9 and Supporting Information S1**). However, since cell cycle genes were over-represented in the 19 TSGs selected, and upon further examination several additional cyclin dependent kinase inhibitors (CDKIs) could be identified immediately below our threshold (data not shown), we further focused on CDKIs p21, p27 and p15.

**Figure 6:**
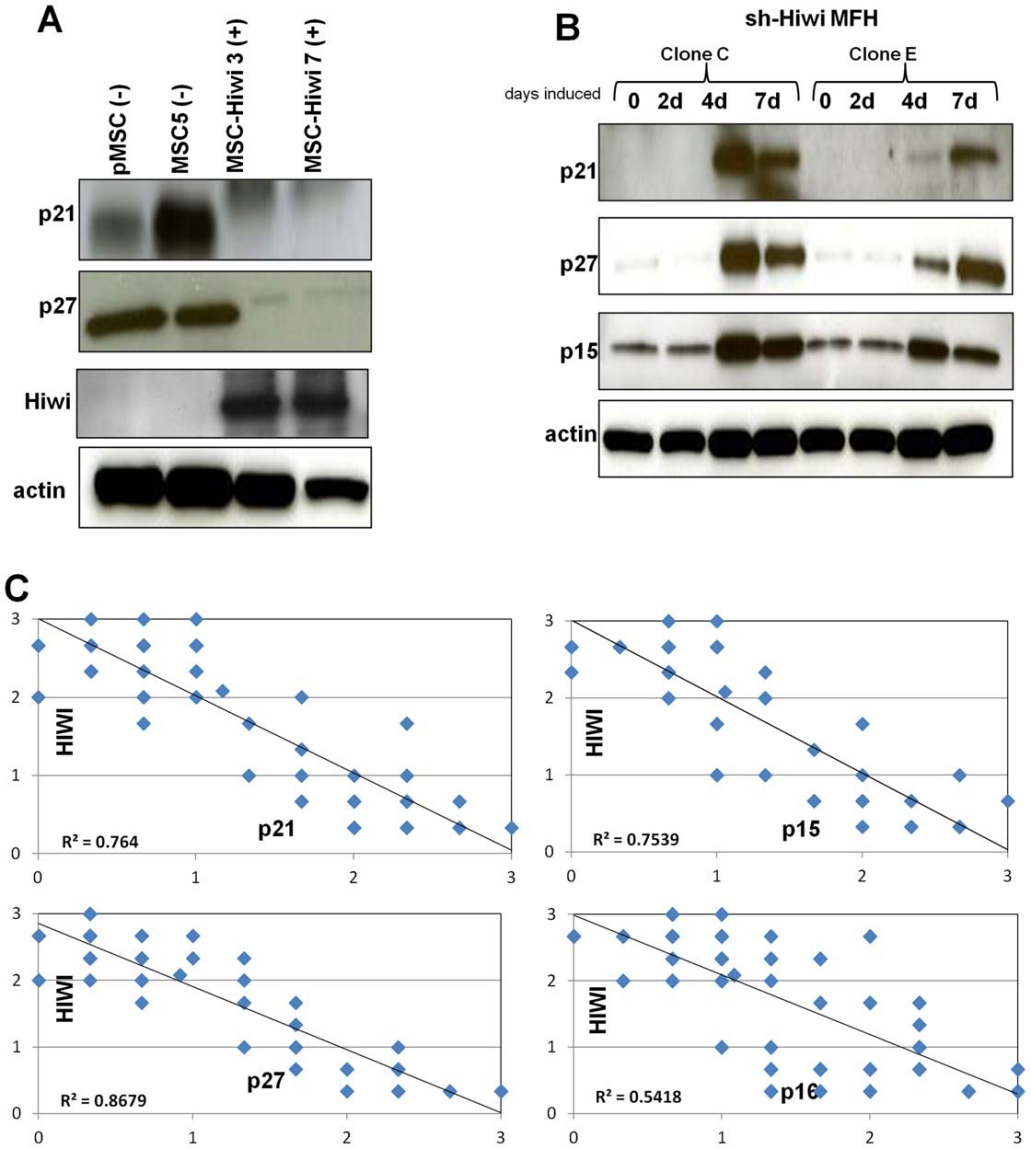
CDKIs are decreased in Hiwi–MSCs. (A) Western blot of indicated proteins reveals that p21 and p27 are decreased in Hiwi-MSCs. (B) Western blot of indicated proteins reveals that p21, p27 and p15 are increased in sh-Hiwi MFH cells that have been induced with doxycycline for 4 or 7 days. (C) IHC analysis of a human sarcoma TMA reveals a tight inverse correlation of p21, p27 and p15 expression (x-axes) to Hiwi expression (y-axis) (R^2^ = 0.764; R^2^ = 0.8679; R^2^ = 0.7539, respectively) but no such correlation for p16 expression (x-axis) to Hiwi expression (y-axis) (R^2^ = 0.5418). Ten cases of each subtype (present in triplicate) were scored from 0 to 2 blindly by sarcoma pathologists. Average scores for each case are plotted here.

We validated the above CDKIs as responsive at the protein level to Hiwi. CDKIs decrease in Hiwi-MSCs and increase in sh-Hiwi MFH upon doxycycline induction (**Figures 6A, B**). We further performed IHC on the human sarcoma tissue microarray used to assess Hiwi (**Figure 1A**); and show that p15, p21, and p27 show a tight IHC-based inverse correlation to Hiwi levels (**Figure 6C**). Ten cases of each subtype (present in triplicate) were scored from 0 to 2 blindly by sarcoma pathologists for each of the indicated proteins. Average scores are plotted here. Importantly, another CDKI, p16, which was not identified in our screen, does not show such a correlation at the IHC level. Previous analyses have only inversely linked p27 to sarcoma grade [28]. Thus our demonstration of an inverse relationship to Hiwi for p15, p21 and p27, combined with our functional data, leads us to conclude that our observations in model systems apply to human sarcomas. Based on the latter data, although we cannot absolutely exclude that DNA methylation is occurring at a non-canonical CpG sites, the lack of any detectable methylation via sequencing suggests to us that DNA-methylation is not occurring at the promoters of Hiwi associated DNA-methylation dependent genes.

## Discussion

Using primary mesenchymal stem cells, transgenic mouse models and human tumor samples we show here that: (1) Hiwi is directly tumorigenic; (2) Hiwi-expressing tumors may be addicted to Hiwi expression; (3) Hiwi mediated tumorigenesis is associated with global DNA-hypermethylation and is reversible using DNA-methyltransferase inhibitors; (4) Hiwi associated global DNA-hypermethylation occurs at non-promoter CpG regions; and (5) Hiwi levels correlate inversely with levels of known tumor suppressor genes. To the best of our knowledge, this is the first mechanistic examination of Hiwi functionality in a mammalian cancer context. Our studies reveal not only a novel oncogenic role for Hiwi as a driver of tumorigenesis, but also that the use of epigenetic agents may be clinically beneficial for treatment of tumors that express Hiwi. Additionally, our data show that Hiwi-mediated methylation is associated with DNA hyper-methylation with subsequent genetic and epigenetic changes that favor a tumorigenic state, reconciling the outstanding conundrum of how Hiwi may act appropriately to promote genomic integrity during early development (via transposon silencing) and inappropriately in adult tissues with subsequent tumorigenesis.

Our data suggest that Hiwi is directly tumorigenic in multiple assays, although a direct genetic basis for Hiwi up-regulation in cancer is still lacking. We have assayed for Hiwi chromosomal amplification using the sarcoma tissue microarray and correlated Hiwi IHC-based expression to Hiwi chromosomal amplification. Only one case out of 45 examined carried a true chromosomal amplification of the Hiwi locus on chromosomal 12 (**Figure S12 and Supporting Information S1**). Hiwi cDNA has been independently isolated from several human cancers and developing sperm [11], [13] without sequence divergence; thus although we cannot rule out a stabilizing mutation, it seems unlikely. Since part of the tumorigenic process involves a recapitulation of the embryonic state, similar mechanisms that up-regulate Hiwi during embryological development may result in Hiwi's up-regulation in cancer.

Despite the tight correlations between Hiwi expression, global DNA methylation, and tumorigenesis provided herein; several pieces still need to be elucidated. In our models and others [29], [30] Hiwi is predominantly cytoplasmic. Furthermore, the protein-level up-regulation of DNMT1, DNMT3a and MDB2 (**Figure 2D**) is post-transcriptional (RNA levels do not change in either Hiwi-MSCs compared to parental cells or in sh-Hiwi MFH before and after doxycycline induction, data not shown). These data together, and given previous reports of Hiwi's interaction with ribosomes [31] and its cytoplasmic localization, Hiwi-associated DNA-methylation may involve as yet unidentified control of DNMT translation. The exact relationship between Hiwi and its mechanistic epigenetic control is an ongoing endeavor both in our laboratory within a cancer context and in the laboratories of many others interested in the role of Piwi proteins in development [1], [2], [3], [4], [5], [6], [7], [8], [9].

Even in mice where Piwi orthologs have been more extensively studied in terms of DNA methylation [7], [8] the exact mechanism by which Piwi family members mediate DNA methylation during normal embryological development is still unclear. Our data that (1) Hiwi translationally up-regulates DNMTs, (2) global DNA methylation (at CpG and non-CpG sites) correlate directly with Hiwi levels, (3) promoter CpG methylation does not change during Hiwi down-regulation and (4) Hiwi down-regulation and 5-azacytidine treatment are mechanistically similar, taken all together, suggest that Hiwi-associated DNA-methylation is occurring globally at non-CpG promoter areas and/or at repetitive element regions. Our inability to detect any DNA promoter methylation via sequencing at either the Line1 element or at the Rb promoter (two transcripts heavily regulated by Hiwi) leads us to suggest that promoters are not methylated. DNA methylation at repetitive element regions has been previously reported to result in long distance gene silencing via chromatin remodeling [32], [33] and given previous reports that Piwi proteins associate with small RNAs (piRNAs) with homology to repetitive elements [10], [34] may explain the lack of gene promoter methylation observed in our systems. On this note a recent report by Sugimoto et al [35] found that another Hiwi family member, Hiwi2, induces p16 silencing via histone 3 lysine 9 methylation, but not, however, gene promoter DNA methylation. To examine the possibility of Hiwi-mediated histone methylation changes, we performed immunofluorescence staining for various histone marks (**Figure S13A–F**) on our dox-ind-sh-Hiwi MFH cells. However, we were not able to discern any alterations in any histone marks examined during Hiwi down-regulation, although we do not doubt that just as reported by Sugimoto et al, promoters of silenced genes will be associated with silencing chromatin marks. Regardless, we believe that stringent statistical cut-offs and the overlapping of gene lists from several independent analyses, clones, and model organisms, resulted in a gene list that likely belies the true extent of gene silencing found in Hiwi expressing cancers. The identified genes are likely to be extremely specific and serve as the basis for further studies of Hiwi mediated gene regulation, tumorigenesis and DNA methylation.

Our observation that Hiwi-mediated tumorigenesis is associated with increased global DNA methylation is somewhat discordant from the more widely accepted notion that global methylation levels are decreased in a variety of human cancers despite specific increase in both promoter and non-promoter CpG island methylation. Thus the global hypomethylation observed in cancer is believed to predominantly occur via repetitive element hypomethylation (which accounts for a significant portion of the human genome; reviewed extensively in [36], [37]). As a result of its inherent developmental preference for repetitive element silencing via DNA methylation, Hiwi may preferentially target repetitive elements for methylation in cancer cells thus mechanistically accounting for an exception to the commonly observed global hypomethylation of cancer.

In conclusion, numerous recent reports of high levels of Hiwi in all cancers examined have raised important questions about the role of Hiwi in adult neoplastic tissues and seem to contradict its known roles in maintaining genome integrity in both germline and somatic stem cells. The data presented here are, to our knowledge, the first to define a causative oncogenic role for Hiwi in human cancer and to elucidate that DNA methylation dependent silencing of tumor suppressor genes accounts for the tumorigenesis. In doing so, we not only reconcile Hiwi's genomic protective and tumorigenic properties but also provide a therapeutic rationale for treating patients with Hiwi-expressing tumors epigenetically by means of DNA-methyltransferase inhibitors.

## Methods

### Colony formation assays

Cells were suspended in 0.3% agar (Sigma) in culture medium and plated into 6 well plates, with a base layer of 0.6% agar in culture medium. For doxycyline and 5-azacytidine treatments, cells were pre-treated for 7 days in culture before beginning colony formation assay. Cells were kept in drug-spiked media during the assay and monitored for colony formation. Pictures were taken after 4 weeks. Experiments were performed 2 times, each time in duplicate.

### Quantitative and semi-quantitative RT PCR

Total RNA was extracted from the indicated cell lines using RNeasy RNA extraction kit (Qiagen) according to the manufacturer's protocol. 1 µg of RNA was transcribed into cDNA using Super-Script III First Strand Synthesis System for RT-PCR (Invitrogen). To assess the expression levels of IAP, Line1, and β-actin control semi-quantitative RT-PCR reactions containing Platinum Blue PCR mix (Invitrogen), 1 ul cDNA and corresponding primers were run at the following PCR program: 95°C×2 min; 95°C×30 s, 55°C×30 s, 68°C×45 s for 28 cycles; 68×5 min. Quantitative RT-PCRs were performed by the Taub Core Facility (CUMC). Sequences for IAP and Line1 primers for both semi-quantitative and quantitative RT-PCRs, from Aravin et al [16], were as follows: Line1 forward: 5′ GAGAACATCGGCACAACAATC; Line1 reverse: 5′ TTTATTGGCGAGTTGAGACCA; IAP forward: 5′ CAGACTGGGAGGAAGAAGCA; IAP reverse: 5′ ATTGTTCCCTCACTGGCAAA. Experiments were performed in triplicate.

### Assessment of DNA methylation levels

Genomic DNA was isolated by DNeasy Blood and Tissue Kit (Qiagen). Global DNA methylation levels were assessed by Methylflash Methylated DNA Quantification Kit (Epigentek) and read on a plate reader at 490 nm, according to manufacturer's protocol. Experiments were performed in triplicate.

### Immunohistochemistry (IHC)

IHC was performed as previously described by us [17]. Briefly, formalin-fixed, paraffin-embedded (FFPE) tissues were rehydrated and treated with citric buffer for antigen retrieval. Slides were blocked with 10% horse of goat serum in 2% BSA-PBS and then incubated in primary antibody (diluted in 2% BSA-PBS) overnight at 4°C. Following 30 minutes of secondary antibody and tertiary antibody (Vector Labs) incubation, slides were developed with 3,3-Diaminobenzidine (DAB) and counterstained with Hematoxyalin. For cells, fixation was performed with 50% Methanol/Acetone for 10 minutes, then blocking, primary antibody incubation, and detection was performed as for FFPE IHC. Primary antibodies used in these studies: Hiwi (Abcam, 12337); p15 INK4b (Novus Biologicals, NB100-91906); p16 (BD Pharmingen, G175-405); p21 (Santa Cruz, sc-6246); p27 (Santa Cruz, sc-528).

### Sarcoma tissue microarray (TMA)

The TMA contained 10 cases of each sarcoma subtype in triplicate. For TMA analysis, IHC staining was scored on a scale of 0 to 2 by multiple pathologists. The average score for each sarcoma subtype was calculated and representative pictures are shown.

### Western blotting

Standard western blotting technique was used. Briefly, protein lysate was collected from cells with RIPA buffer (Boston BioProducts) and 50 ug were run on a 4–20% Tris-glycine gradient gel (Invitrogen). Nitrocellulose membranes were incubated overnight at 4°C in primary antibody: DNMT1 (1∶500, abcam 92453); DNMT3a (1∶500, abgent AP1034a); MDB2 (1∶1000, abcam 38646); Hiwi (1∶500 ProSci 45-735P).

### Bone and fat differentiation

Bone or fat differentiation was assessed in MSCs after 21 days in bone or fat differentiation media, as previously published [18]. Approximate percentage of differentiated cells was calculated based on the average alizarin red s or oil red o staining over 3 independent experiments.

### Ethics statement

All mouse experiments for this specific study were approved by the Columbia IRB as described under protocol AAAA9669 and in accordance with Columbia University Animal Welfare and IUCAC policy.

### Xenograft generation

NOD-SCID mice were subcutaneously injected, in triplicate, with 1 million cells of each indicated cell type, as described previously [1]. Tumor formation was monitored for 5 weeks and mice were sacrificed when tumor size reached 1 cm, in accordance with Columbia University Animal Welfare and IUCAC policy under IRB protocol AAAA9669.

### Generation of Hiwi expressing mesenchymal stem cells (Hiwi-MSCs) and inducible Hiwi knock down MFH cells

Details on cell lines, growth conditions, differentiation induction and assessment have been previously described by us [17], [19]. Hiwi-MSCs were generated by cloning Hiwi cDNA (gift of Dr. G. Hannon) into the pLENTI vector (Invitrogen). Transfection into 293FT cells/infection into MSCs was performed with the ViraPower system (Invitrogen). Doxycycline-inducible short hairpin Hiwi-MFH cells were generated by transfection of lentiviral pTRIPZ Hiwi short hairpin vector (OpenBiosystems) into 293FT cells and then infection into MFH cells. 0.5 ug/ml doxycycline in growth media was used to induce sh-Hiwi expression. sh-Hiwi constructs are 3 separate shRNA plasmids against Hiwi, pooled and co-transfected into MFH cells.

### Generation of Prx1-Hiwi transgenic mice

The human Hiwi gene was subcloned under the control of the Prx1 promoter [20], [21] (gift of Dr. C. Tabin). Founders with highest transgene expression were chosen for further analysis.

### Gene expression profiling

RNA from the indicated cell lines were hybridized to Affymetrix HG U133 (human) or Affymetrix 430 2.0 (mouse) oligonucleotide arrays per standard protocols of the Columbia Genomics Core Facility. Class-comparison analysis using two-sided Student t-tests identified mRNAs that were differentially expressed between indicated samples (p<0.05). Raw data will be deposited in the public repository.

### Promoter methylation profiling

Promoter methylation profiling was done using Illumina Meth27 promoter arrays. Samples were run at Roswell Park Cancer Institute Genomics Facility. Average beta values, corresponding to amount of methylation, were then analyzed using Genespring software.

For additional information please see Supplementary Methods in Supporting Information S1.

### Bisulfite quantitative PCR

Bisulfite quantitative PCRs for Line1 and IAP on Hiwi-MSCs were performed at the Taub Institute Core Facility (CUMC). Briefly, Hiwi-MSCs were treated for 18 h with the indicated concentration of 5-azacytidine and then DNA was collected. After quantitative PCR for either Line1 or IAP, the resulting PCR amplicon was bisulfite converted to assess methylation in that region. Primers for IAP and Line1 from Lane et al [22], were as follows: Line1 forward: 5′ GTTAGAGAATTTGATAGTTTTTGGAATAGG; Line1 reverse: 5′CCAAAACAAAACCTTTCTCAAACACTATAT; IAP forward: 5′TTGATAGTTGTGTTTTAAGTGGTAAATAAA; IAP reverse: 5′AAAACACCACAAACCAAAATCTTCTAC.

## Supporting Information

Supporting Information S1 Supporting Information includes supporting text further explaining data in supporting figures, supporting methods used in this study, and supporting references.
(DOC)

Figure S1 **Hiwi is highly expressed in human undifferentiated sarcoma samples.** Immunohistochemical (IHC) analysis of Hiwi on a human sarcoma tissue microarray (TMA). Ten cases of each subtype (present in triplicate) were scored from 0 to 2 blindly by sarcoma pathologists. HGUS =  high grade undifferentiated sarcoma. Average scores are plotted here for each subtype. Error bars represent standard error ** = p<0.005 by Student's T-Test
(TIF)

Figure S2 **Validation of Hiwi-MSCs.** (A) Parental MSCs, MSC5 (a clone which is selection-marker resistant but doesn't express Hiwi), and Hiwi–expressing clones 3 and 7 were analyzed by quantitative RT-PCR for Hiwi expression. MSC-Hiwi 7 was arbitrarily set at 1. (B) MSC clones were analyzed by Western Blot for Hiwi expression levels. MSC-Hiwi 3 and 7 were positive clones, where as MSC2, 5, and 6 gained selection marker resistance without expressing Hiwi. MSC5 was chosen for further experiments. AGS and N87 gastric cancer cell lines have been previously reported as positive controls for Hiwi expression. See Supporting Information S1 for further explanation of non-specific bands.
(TIF)

Figure S3 **Hiwi-expressing MSCs form large tumors in a xenograft model.** Quantification of average xenograft size in grams, after 5 weeks of monitoring. Xenografts were diagnosed histo-pathologically to be high grade undifferentiated sarcomas. Error bars represent standard error and xenografts were performed in triplicate. * = p<0.05 by Student's T-Test
(TIF)

Figure S4 **Hiwi and Ras cooperate to become highly oncogenic in MSCs.** MEFs (left column) or MSCs (right column) were transfected with either Hiwi alone, Ras alone or with both Hiwi and Ras and then put into colony formation assays. At 4 weeks in colony formation assay, both Hiwi alone and Ras alone formed colonies in MSCs (p<0.05 compared to untransfected control) and together they formed significantly more colonies (p<0.005 compared to untransfected control). No significant changes in colony formation were observed in the transfected MEFs (p>0.5 for all transfections compared to untransfected control). All experiments were performed in triplicate. Representative pictures are shown here.
(TIF)

Figure S5 **Treatment of Hiwi-MSCs with 5-azacytidine can reverse Hiwi-mediated transposon silencing.** (A) Semi-quantitative RT-PCR for Line1 and IAP transposon expression on Hiwi-MSCs treated 18 h with the indicated concentration of 5-azacytidine. Actin is a loading control. Experiments were performed in triplicate. (B) Semi-quantitative RT-PCR for Line1 and IAP transposon expression on Hiwi-MSCs treated 18 h with 50 uM of 5-azacytidine or 5-aza-2-deoxycytidine. Actin is a loading control. Experiments were performed in triplicate.
(TIF)

Figure S6 **Expression of Hiwi in sarcoma cell lines.** (A) Quantitative RT-PCR of Hiwi in a panel of human sarcoma cell lines reveals that MFH has high Hiwi RNA levels. (B) Immunohistochemical analysis of Hiwi in a panel of human sarcoma cell lines reveals that MFH has high Hiwi protein levels.
(TIF)

Figure S7 **Hiwi down-regulation and 5-azacytidine treatment are mechanistically similar.** (A) Venn diagram of overlapping differentially expressed genes in both sh-Hiwi MFH cells and in 5-azacytidine-treated MFH cells. At early time points, over 75% of differentially expressed genes following Hiwi down-regulation are also differentially-expressed during 5-azacytidine treatment. 99% of the overlapping genes move in the same direction in both conditions. (B) At late time points, over 50% of differentially expressed genes following Hiwi down-regulation are also differentially-expressed during 5-azacytidine treatment. 93% of the overlapping genes move in the same direction in both conditions
(TIF)

Figure S8 **Genes with CpG site promoter hypo-methyation in sh-Hiwi MFH cells.** Analysis of Illumina Meth27 promoter methylation arrays reveals only 18 CpG sites that show at least a 10% decrease in methylation after 7 days of doxycycline treatment of sh-Hiwi MFH cells. Genes to which the CpG sites belong are identified here.
(TIF)

Figure S9 **CpG methylated genes do not correlate to genes expression changes in sh-Hiwi MFH cells and Hiwi MSCs.** (A) Gene expression profiles of the 17 identified genes with CpG site hypo-methylation, after 0, 2, 4 or 7 days of doxycycline induction of sh-Hiwi MFH cells. While corresponding CpG sites are hypo-methylated, there is no corresponding increase in gene expression. (B) Gene expression profiles of the 17 identified genes with CpG site hypo-methylation in Hiwi-MSCs. Conversely, there is no decrease in expression of these genes. Because each of the 17 identified genes contains multiple spots on the array, corresponding to multiple Gene IDs, multiple rows for each gene are shown in both (A) and (B).
(TIF)

Figure S10 **Methylation of Rb1 and Line1 promoter CpG islands do not change as Hiwi levels change.** Bisulfite sequencing of Rb1 promoter CpGs in Hiwi-MSCs (top graph) and in sh-Hiwi MFH cells (middle graph) reveal no methylation changes as Hiwi levels change. Similarly, bisulfite sequencing of Line1 CpGs in Hiwi-MSCs (bottom graph) reveal no methylation changes as Hiwi levels change.
(TIF)

Figure S11 **IAP and Line1 transposon methylation is unchanged in Hiwi-MSCs.** Quantitative PCR of IAP or Line1 transposon expression, followed by bisulfite conversion, on parental MSCs, MSC5, Hiwi-MSC3 and Hiwi-MSC7 treated with the indicated concentration of 5-azacytidine for 18 h. Experiments were performed in triplicate. Error bars represent standard error.
(TIF)

Figure S12 **Hiwi is not chromosomally amplified in Hiwi-expressing sarcomas.** DNA FISH was performed on the human sarcoma tissue microarray, using a probe against the Hiwi locus on chromosome 12. (i) Analysis of the sarcomas reveals only 1 out of 45 cases that has a true amplification of Hiwi, a dedifferentiated liposarcoma. (ii) A few cases (5 out of 45) have a copy number increase of Hiwi. (iii) However, the majority of cases (39 out of 45), including all HGUS Hiwi-expressing cases, have no chromosomal amplification of Hiwi.
(TIF)

Figure S13 **Epigenetic histone marks are unchanged in doxycycline-induced sh-Hiwi MFH cells.** Immunofluorescence on sh-Hiwi MFH cells, either uninduced or induced for 7days with doxycycline to knock down Hiwi levels, for the following histone 3 lysine or arginine marks (A) H3K4me; (B) H3K4me; (C) H3K27me; (D) panH3Rme2; (E) H3R2me; (F)H3R17me.
(TIF)

Table S1 Promoter methylation in dox-ind-sh-Hiwi MFH cells does not change compared to uninduced control MFH cells. Raw beta values from the Illumina promoter meth27 arrays are shown here. Beta values are between 0 and 1, corresponding to unmethylated and completely methylated, respectively.
(XLS)
