# Supplementary material for: Hiwi Mediated Tumorigenesis Is Associated with DNA Hypermethylation
Source: PLoS One. 2012 Mar 16;7(3):e33711. doi: 10.1371/journal.pone.0033711 (PMC3306289; doi:10.1371/journal.pone.0033711)
Supplement: Supporting Information S1 — Supporting Information includes supporting text further explaining data in supporting figures, supporting methods used in this study, and supporting references. (DOC) [file pone.0033711.s001.doc]

**Hiwi mediated tumorigenesis is Associated with DNA hypermethylation**

**Supporting Information**

Sara Siddiqi, Melissa Terry, Igor Matushansky

Herbert Irving Comprehensive Cancer Center, Columbia University

**Supporting Text**

**Hiwi is highly expressed in undifferentiated human sarcoma subtypes**

Hiwi protein expression was examined on a variety of human sarcoma subtypes via immunohistochemistry (IHC) on a sarcoma tissue microarray (TMA). Hiwi was found to be expressed at significantly higher levels in undifferentiated sarcoma subtypes as compared to more differentiated subtypes (**Figure S1**). Ten cases of each sarcoma subtype (present in triplicate) were scored from 0 to 2 blindly by sarcoma pathologists. Scores were averaged and are plotted here.

**Generation of Hiwi-MSCs.**

Using a lentiviral construct, we exogenously over-expressed Hiwi in MSCs. In order to validate our Hiwi-MSCs beyond IHC staining, we performed a quantitative RT-PCR (**Figure S2A**) on parental MSCs, MSC5 (a non Hiwi-expressing clone), and Hiwi-MSCs 3 and 7 (Hiwi-expressing clones), along with their corresponding no reverse transcriptase controls. Hiwi-MSC 3 and 7 express high levels of Hiwi, as compared to parental MSCs and MSC5. Additionally, we performed western blotting analysis of various clones (**Figure S2B**). MSC2, 5 and 6, along with parental MSCs, showed no Hiwi expression. Hiwi-MSC clones 3 and 7 express Hiwi (middle band; 100kDa). AGS and N87 gastric cancer cell lines have been previously reported to express Hiwi and were used as positive controls. However, Hiwi is more readily detected in N87 at this exposure, and both are expressed at much lower levels compared to either of our Hiwi-expressing clones. A high non-specific band at about 115kDa is consistently seen in our Western blots with this antibody. Additionally, a lower band around 82kDa is seen in the Hiwi-expressing samples (clones 3 and 7). This band may represent a degradation product of Hiwi, however its exact identity is unclear and we are continuing studies on this possibility.

**Hiwi-MSCs form tumors in vivo**

To test whether Hiwi expression can generate sarcomas in vivo, we subcutaneously injected Hiwi-MSCs into NOD-SCID mice, in triplicate. Xenograft formation was monitored for 5 weeks. We found that Hiwi-MSCs formed large high grade undifferentiated sarcomas in our xenograft model, as diagnosed blindly by sarcoma pathologists (see acknowledgements). These xenograft weights were quantified (**Figure S3**) and xenografts derived from Hiwi-MSCs were significantly larger than small calcified nodules found in parental MSCs and in a non-Hiwi expressing clone, MSC 5.

**Hiwi is an Oncogene**

To further explore the oncogenic properties of Hiwi we performed classical oncogene cooperation assays in which Hiwi was infected into MSCs and MEFs alone and in conjunction with Ras. Hiwi-transformed MSCs form large colonies, which significantly increase in size and frequency when combined with Ras (**Figure S4**). Interestingly we did not observe a similar transformative effect using MEFs suggesting that Hiwi's function may be limited to stem cells; in agreement with its known developmental role. These results indicate that Hiwi is sufficient to transform progenitors into sarcomas.

**Treatment of Hiwi-MSCs with either 5-azacytidine can reverse Hiwi-mediated transposon silencing.**

We show that treatment of Hiwi-MSCs with either 5-azacytidine or 5-aza-2-deoxycytidine can reverse Hiwi-mediated transposon silencing (**Figure S5**). 5-azacytidine treatments at lower concentrations (5uM or 10uM) show some restoration of Line1 transposon expression (**Figure S5A**, red box). However, a higher concentration of 5-azacytidine treatment (50uM) is able to restore both IAP and Line1 transposon expression to levels comparable with parental MSCs, best seen for MSC-Hiwi7 (**Figure S5B**, red box) which has higher baseline Hiwi expression (**Figure S2A**) and is more tumorigenic (**Figure S3**). No such changes in transposons were observed in non-Hiwi expressing cells (**Figure S5B,** left sided lanes). Treatment of Hiwi-MSC3 with both 5-azacytidine and 5-aza-2-deoxycytidine was highly toxic to the cells, despite our repeated treatment attempts.

**Identification of a human sarcoma cell line expressing endogenous high Hiwi levels.**

We have previously characterized a panel of human sarcoma cell lines [1]. Previously reported gene expression analysis predicted that the high grade undifferentiated sarcoma cell line, MFH, expressed high levels of Hiwi at the RNA level. We validated Hiwi expression in MFH cells using quantitative RT-PCR (**Figure S6A**). We then assessed Hiwi expression via immunohistochemistry in MFH cells as compared to other sarcoma cell lines (**Figure S6B**). Both RT-PCR and IHC confirm our initial gene expression analysis and identify MFH as a sarcoma cell line that endogenously expresses Hiwi.

**Hiwi down-regulation and 5-azacytidine treatment are mechanistically similar.**

Using differentially expressed gene sets from both doxyclycine-induced sh-Hiwi MFH and 5-azacytidine-treated sh-Hiwi MFH cells, we found that many altered genes overlap in both conditions. At early time points (**Figure S7A**), we find that of the genes differentially expressed during down-regulation of Hiwi, about 75% are also differentially expressed during 5-azacytidine treatment. Moreover, the vast majority (over 99%) of these overlapping genes move in the same direction both gene sets. Similarly, at later time points (**Figure S7B**), about half of the genes differentially expressed during Hiwi down-regulation are also differentially expressed during 5-azacytidine treatment. Of the overlapping genes, about 93% trend similarly in both data sets. These data suggest that Hiwi down-regulation in MFH cells is mechanistically similar to 5-azacytidine treatment of MFH cells.

**Identification of genes showing CpG island hypo-methylation following down-regulation of Hiwi in MFH**.

Using an Illumina Meth27 promoter methylation array, we assessed the methylation levels of 27000 promoter CpG sites in sh-Hiwi MFH cells, before and after 7 days of doxycycline induction. We identified only 18 CpG sites that showed at least a 10% decrease in methylation (**Figure S8**), which map to 17 unique genes. The genes corresponding to those CpG sites are identified here.

**Lack of Correlation of CpG Methylated Genes to Gene Expression Levels.**

We then asked if the methylation changes in the CpG sites of the 17 genes identified in Supplementary Figure 8 correlate with gene expression changes observed in sh-Hiwi MFH cells. Using gene expression arrays (as described in the main text), to identify gene expression changes, we found that the 17 genes associated with these 18 CpG sites did not show a simultaneous increase in gene expression following Hiwi down-regulation (**Figure S9A**) nor did they show a decrease in Hiwi-MSCs as compared to parental MSCs (**Figure S9B**) (Multiple Gene IDs are shown for each gene; see figure legend for details). These data suggest that Hiwi mediates DNA methylation at non-CpG sites, in agreement with our previous data.

**Bisulfite sequencing of Rb1 and Line1 at known CpG islands confirms lack of CpG methylation.**

Because overlap of gene expression changes in (1) Hiwi-MSCs, (2) doxycycline-induced sh-Hiwi MFH and (3) 5-azacytidine-treated sh-Hiwi MFH yielded 19 genes, one of which was Rb1, we decided to further explore the methylation status of Rb1. We performed bisulfite sequencing of the Rb1promoter in both Hiwi-MSCs and in sh-Hiwi MFH cells (**Figure S10**, top and middle graphs). In agreement with our CpG promoter methyl array results, there are no changes in the methylation status of the Rb1 promoter CpG sites as Hiwi levels change. Additionally, we examined, via bisulfite sequencing, the methylation status of the Line1 transposon (**Figure S10,** bottom graph). We find that these CpG sites also remain unchanged as Hiwi levels change. These data suggest that because no methylation changes occur in these methylation-dependent genes, Hiwi-mediated DNA methylation is non-CpG sites methylation.

**Bisulfite quantitative PCRs of Line1 and IAP confirm no significant changes in methylation.**

Although bisulfite sequencing of the Rb1 promoter and of Line1 did not show any changes in CpG methylation in Hiwi-MSCs, we performed additional quantitative PCRs followed by bisulfite conversion on Hiwi-MSCs that were treated with 5uM, 10uM or 50uM 5-azacytidine for 18h (**Figure S11**). There are no significant changes in the methylation of these transposon regions in the Hiwi-MSCs, suggesting that although global DNA methylation increases in Hiwi-MSCs, this is not at IAP and Line1 regions specifically.

**Hiwi is not chromosomally amplified in Hiwi-expressing sarcomas.**

We have assayed for Hiwi amplification using the tissue microarray (**Figure 1A**) and attempted to correlate IHC-based expression to Hiwi amplification. However only one case out of 45 examined carried a true chromosomal amplification of the Hiwi locus on chromosome 12 (**Figure S12i**), a few cases (5/45) had a copy number increase of chromosome 12 (**Figure S12ii**) while the majority of cases (39 out of 45), including all HGUS cases, did not have an amplification of chromosome 12 (**Figure S12iii**).

**Histone marks are not altered during Hiwi downregulation in sh-Hiwi MFH cells**

Previous reports have found that another Hiwi family member, Hiwi2 (Piwil4) mediated silencing of p16 tumor suppressor by increasing histone 3 lysine 9 methylation (see main text for further discussion). To examine the possibility that epigenetic histone marks are altered in our sh-Hiwi MFH cells after Hiwi down-regulation, we performed immunofluoescent staining on sh-Hiwi MFH cells before and after doxycycline induction, examining various histone lysine and histone arginine methylation marks (**Figure S13A-F**). However, none of the histone marks examined were altered after Hiwi down-regulation.

**Supporting Methods**

**Colony Formation Assays**

Cells were suspended in 0.3% agar (Sigma) in culture medium and plated into 6 well plates, with a base layer of 0.6% agar in culture medium. For doxycyline and 5-azacytidine treatments, cells were pre-treated for 7 days in culture before beginning colony formation assay. Cells were kept in drug-spiked media during the assay and monitored for colony formation. Pictures were taken after 4 weeks. Experiments were performed in 2 times, each time in duplicate.

**Semi-quantitative RT PCR**

Total RNA was extracted from the indicated cell lines using RNeasy RNA extraction kit (Qiagen) according to the manufacturer’s protocol. 1 µg of RNA was transcribed into cDNA using Super-Script III First Strand Synthesis System for RT-PCR (Invitrogen). To assess the expression levels of IAP, Line1, and β-actin control semi-quantitative RT-PCR reactions containing Platinum Blue PCR mix (Invitrogen), 1ul cDNA and corresponding primers were run at the following PCR program: 95°C x 2min; 95°C x 30s, 55°C x 30s, 68°C x 45s for 28 cycles; 68x5min. Experiments were performed in triplicate.

**Assessment of DNA methylation levels**

Genomic DNA was isolated by DNeasy Blood and Tissue Kit (Qiagen). Global DNA methylation levels were assessed by Methylflash Methylated DNA Quantification Kit (Epigentek) and read on a plate reader at 490nm, according to manufacturer’s protocol. Experiments were performed in triplicate.

**Immunohistochemistry (IHC)**

IHC was performed as previously described by us[1]. Briefly, formalin-fixed, paraffin-embedded tissues were rehydrated and treated with citric buffer for antigen retrieval. Slides were blocked with 10% horse of goat serum in 2% BSA-PBS and then incubated in primary antibody (diluted in 2% BSA-PBS) overnight at 4°C. Following 30 minutes of secondary antibody and tertiary antibody (Vector Labs) incubation, slides were developed with 3,3-Diaminobenzidine (DAB) and counterstained with Hematoxyalin. For cells, fixation was performed with 50% Methanol/Acetone for 10 minutes, then blocking, primary antibody incubation, and detection was performed as for FFPE IHC. Primary antibodies used in these studies: Hiwi (Abcam, 12337); p15 INK4b (Novus Biologicals, NB100-91906); p16 (BD Pharmingen, G175-405); p21 (Santa Cruz, sc-6246); p27 (Santa Cruz, sc-528).

**Immunofluorescence (IF)**

Standard IF technique was used. Briefly, cells were fixed in cold 1:1 Methanol-Acetone and blocked with 10% donkey serum in 2% BSA-PBS. The following primary antibodies were applied overnight at 4°C at 1 to 200 dilution in blocking buffer: H3K4me (abcam #ab8898); H3K4me (abcam #ab6000 ); H3K27me (abcam #ab6002); panH3Rme2 (abcam #ab413); H3R2me (abcam #ab80075); H3R17me (abcam #ab8284).

**Sarcoma Tissue Microarray (TMA)**

The TMA contained 10 cases of each sarcoma subtype in triplicate. For TMA analysis, IHC staining was scored on a scale of 0 to 2 by multiple sarcoma pathologists. The average score for each sarcoma subtype was calculated and representative pictures are shown.

**Western Blotting**

Standard western blotting technique was used. Briefly, protein lysate was collected from cells with RIPA buffer (Boston BioProducts) and 50ug were run on a 4-20% Tris-glycine gradient gel (Invitrogen). Nitrocellulose membranes were incubated overnight at 4°C in primary antibody: DNMT1 (1:500, abcam 92453); DNMT3a (1:500, abgent AP1034a); MDB2 (1:1000, abcam 38646); Hiwi (1:500 ProSci 45-735P).

**Bone and Fat Differention**

Bone or fat differentiation was assessed in MSCs after 21 days in bone or fat differentiation media, as previously published [2]. Approximate percentage of differentiated cells was calculated based on the average alizarin red s or oil red o staining over 3 independent experiments.

**Xenograft Generation**

NOD-SCID mice were subcutaneously injected, in triplicate, with 1 million cells of each indicated cell type, as described previously [1]. Tumor formation was monitored for 5 weeks and mice were sacrificed when tumor size reached 1cm, in accordance with Columbia University Animal Welfare and IUCAC policy under IRB protocol AAAA9669.

**Supporting** **References**

1. Mills, J., et al., *Characterization and comparison of the properties of sarcoma cell lines in vitro and in vivo.* Hum Cell, 2009. **22**(4): p. 85-93.

2. Matushansky, I., et al., *Derivation of sarcomas from mesenchymal stem cells via inactivation of the Wnt pathway.* J Clin Invest, 2007. **117**(11): p. 3248-57.
