# Supplementary figures and images for: Hiwi Mediated Tumorigenesis Is Associated with DNA Hypermethylation
Source: PLoS One. 2012 Mar 16;7(3):e33711. doi: 10.1371/journal.pone.0033711 (PMC3306289; doi:10.1371/journal.pone.0033711)

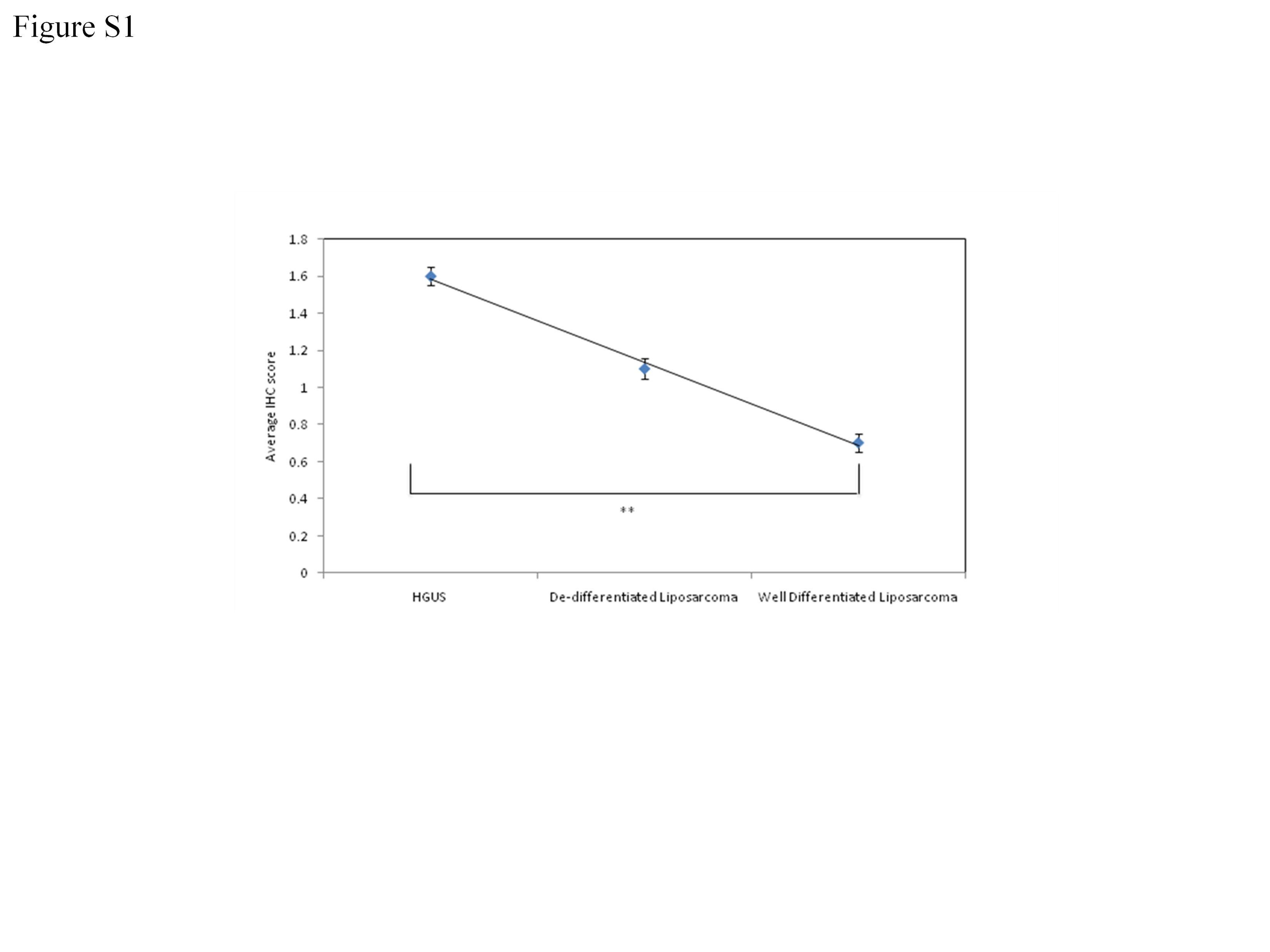

Supplement: Figure S1 — Hiwi is highly expressed in human undifferentiated sarcoma samples. Immunohistochemical (IHC) analysis of Hiwi on a human sarcoma tissue microarray (TMA). Ten cases of each subtype (present in triplicate) were scored from 0 to 2 blindly by sarcoma pathologists. HGUS = high grade undifferentiated sarcoma. Average scores are plotted here for each subtype. Error bars represent standard error ** = p<0.005 by Student's T-Test (TIF) [file pone.0033711.s002.tif]

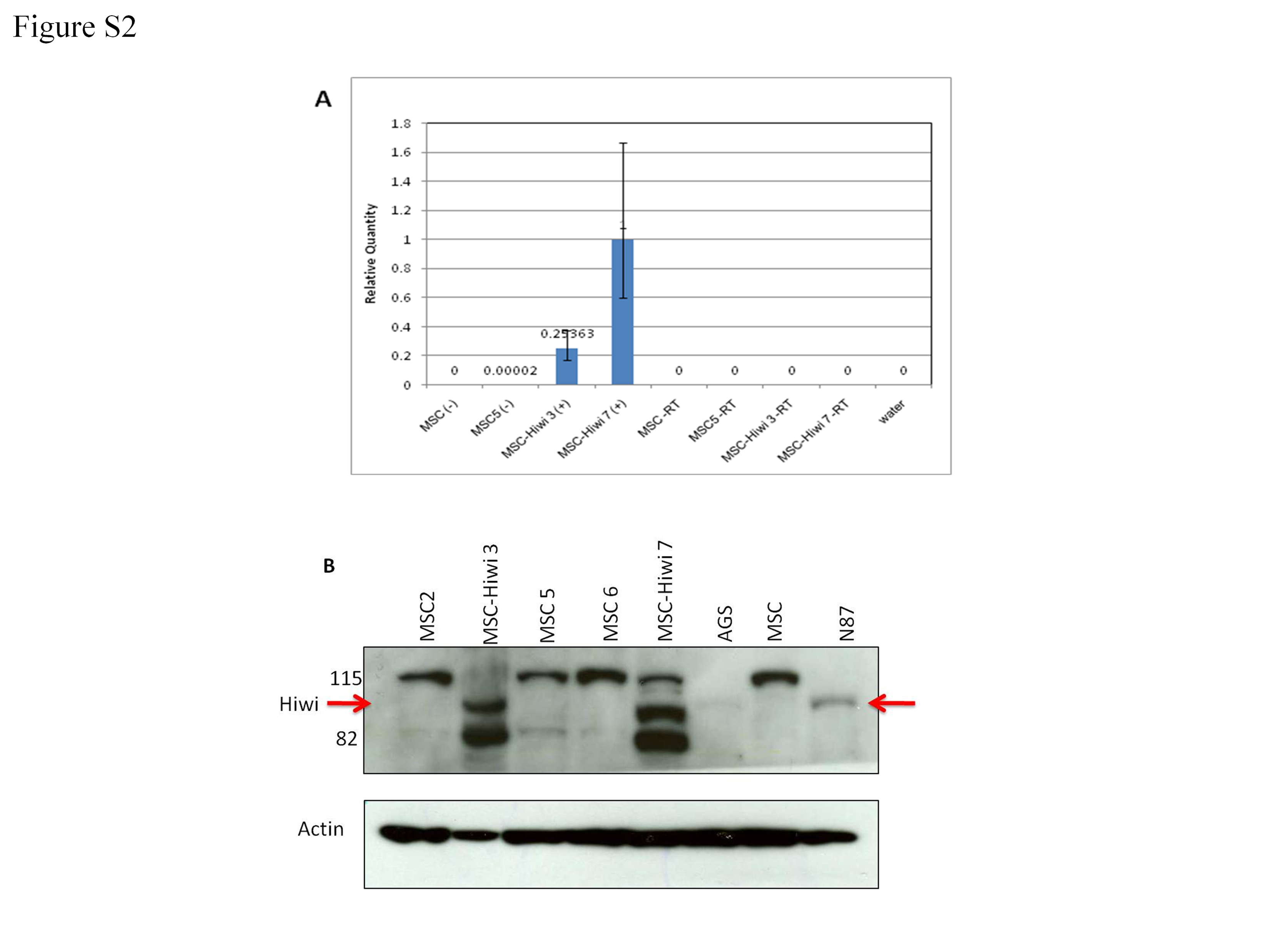

Supplement: Figure S2 — Validation of Hiwi-MSCs. (A) Parental MSCs, MSC5 (a clone which is selection-marker resistant but doesn't express Hiwi), and Hiwi–expressing clones 3 and 7 were analyzed by quantitative RT-PCR for Hiwi expression. MSC-Hiwi 7 was arbitrarily set at 1. (B) MSC clones were analyzed by Western Blot for Hiwi expression levels. MSC-Hiwi 3 and 7 were positive clones, where as MSC2, 5, and 6 gained selection marker resistance without expressing Hiwi. MSC5 was chosen for further experiments. AGS and N87 gastric cancer cell lines have been previously reported as positive controls for Hiwi expression. See Supporting Information S1 for further explanation of non-specific bands. (TIF) [file pone.0033711.s003.tif]

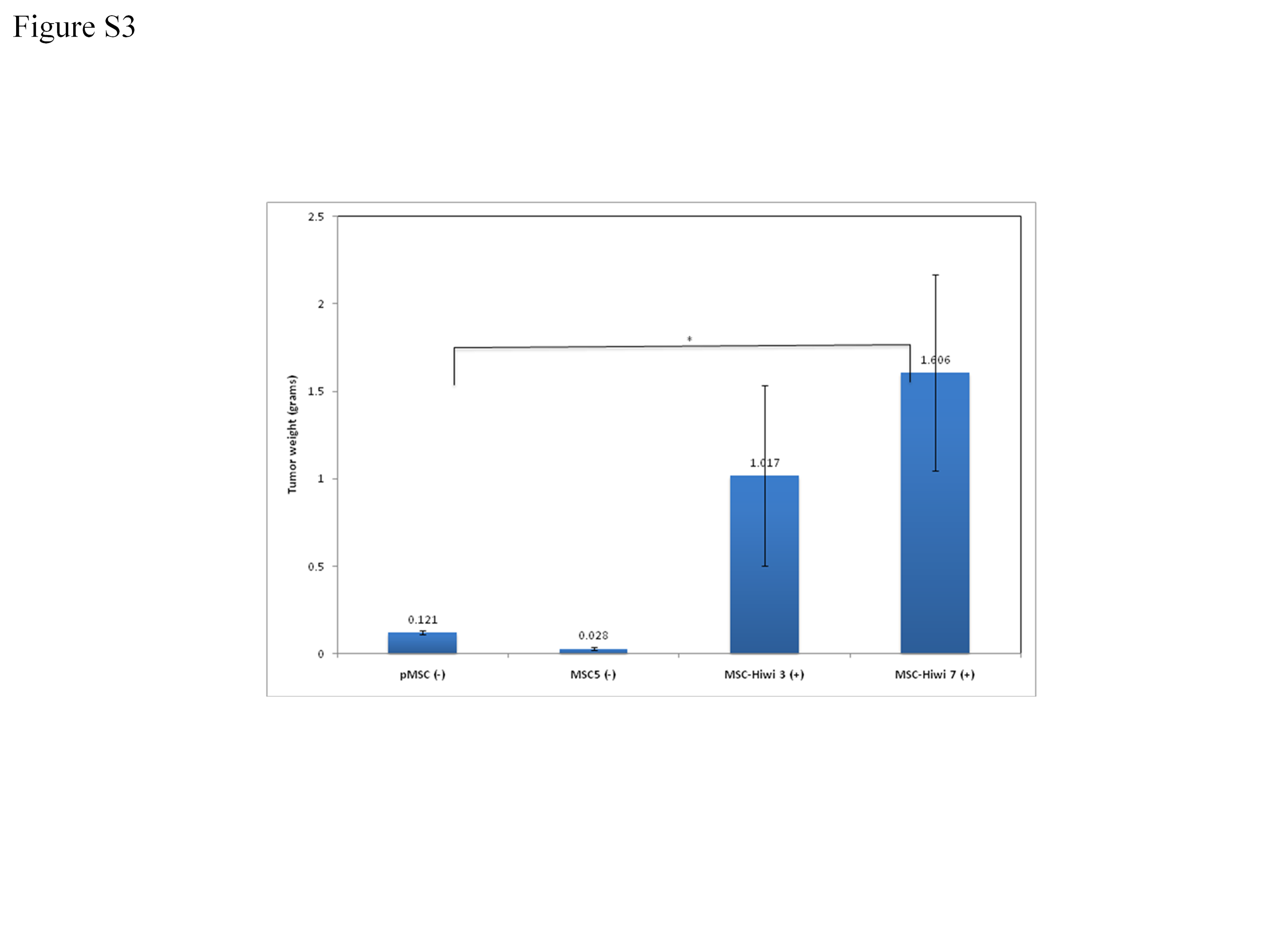

Supplement: Figure S3 — Hiwi-expressing MSCs form large tumors in a xenograft model. Quantification of average xenograft size in grams, after 5 weeks of monitoring. Xenografts were diagnosed histo-pathologically to be high grade undifferentiated sarcomas. Error bars represent standard error and xenografts were performed in triplicate. * = p<0.05 by Student's T-Test (TIF) [file pone.0033711.s004.tif]

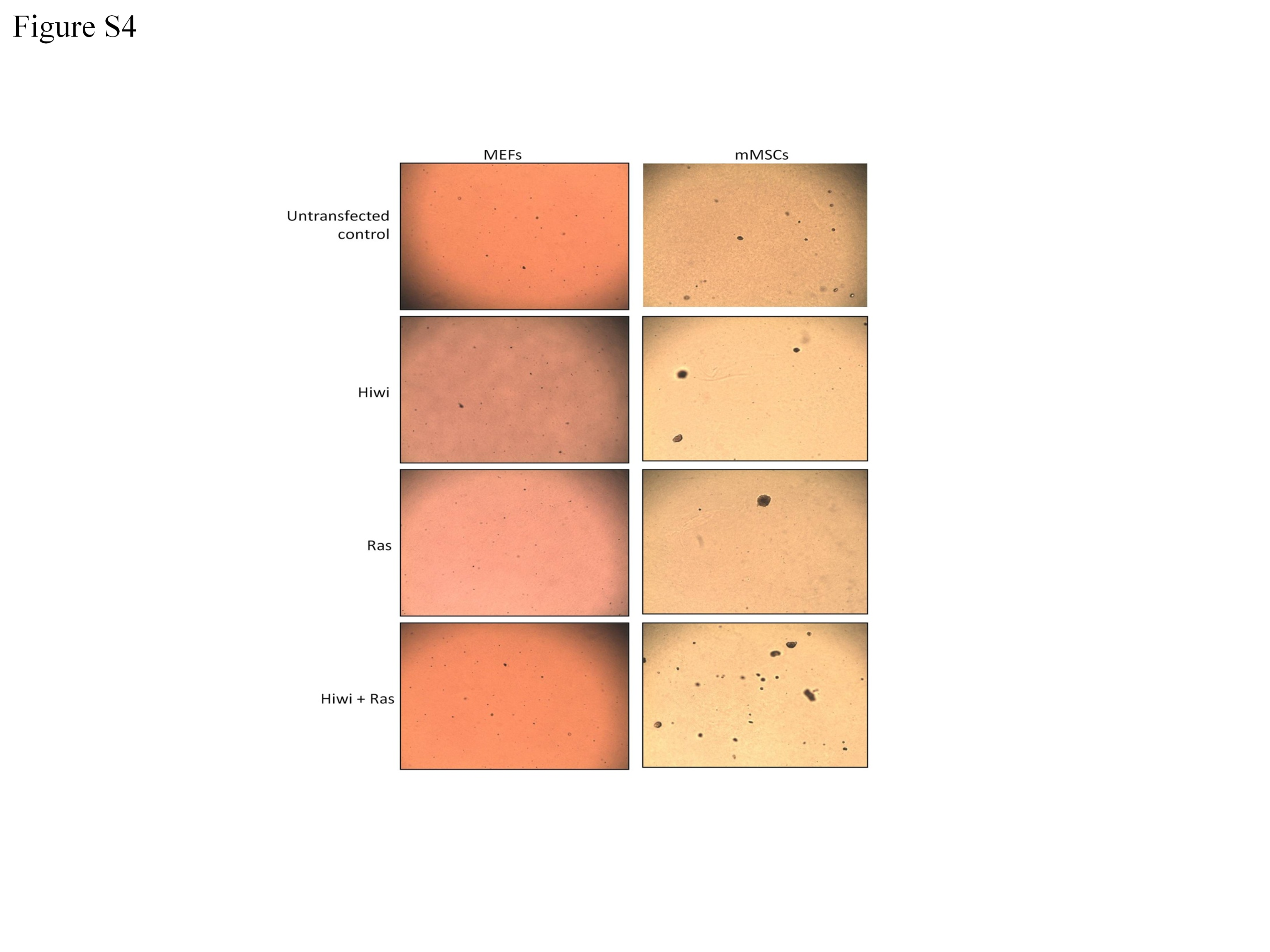

Supplement: Figure S4 — Hiwi and Ras cooperate to become highly oncogenic in MSCs. MEFs (left column) or MSCs (right column) were transfected with either Hiwi alone, Ras alone or with both Hiwi and Ras and then put into colony formation assays. At 4 weeks in colony formation assay, both Hiwi alone and Ras alone formed colonies in MSCs (p<0.05 compared to untransfected control) and together they formed significantly more colonies (p<0.005 compared to untransfected control). No significant changes in colony formation were observed in the transfected MEFs (p>0.5 for all transfections compared to untransfected control). All experiments were performed in triplicate. Representative pictures are shown here. (TIF) [file pone.0033711.s005.tif]

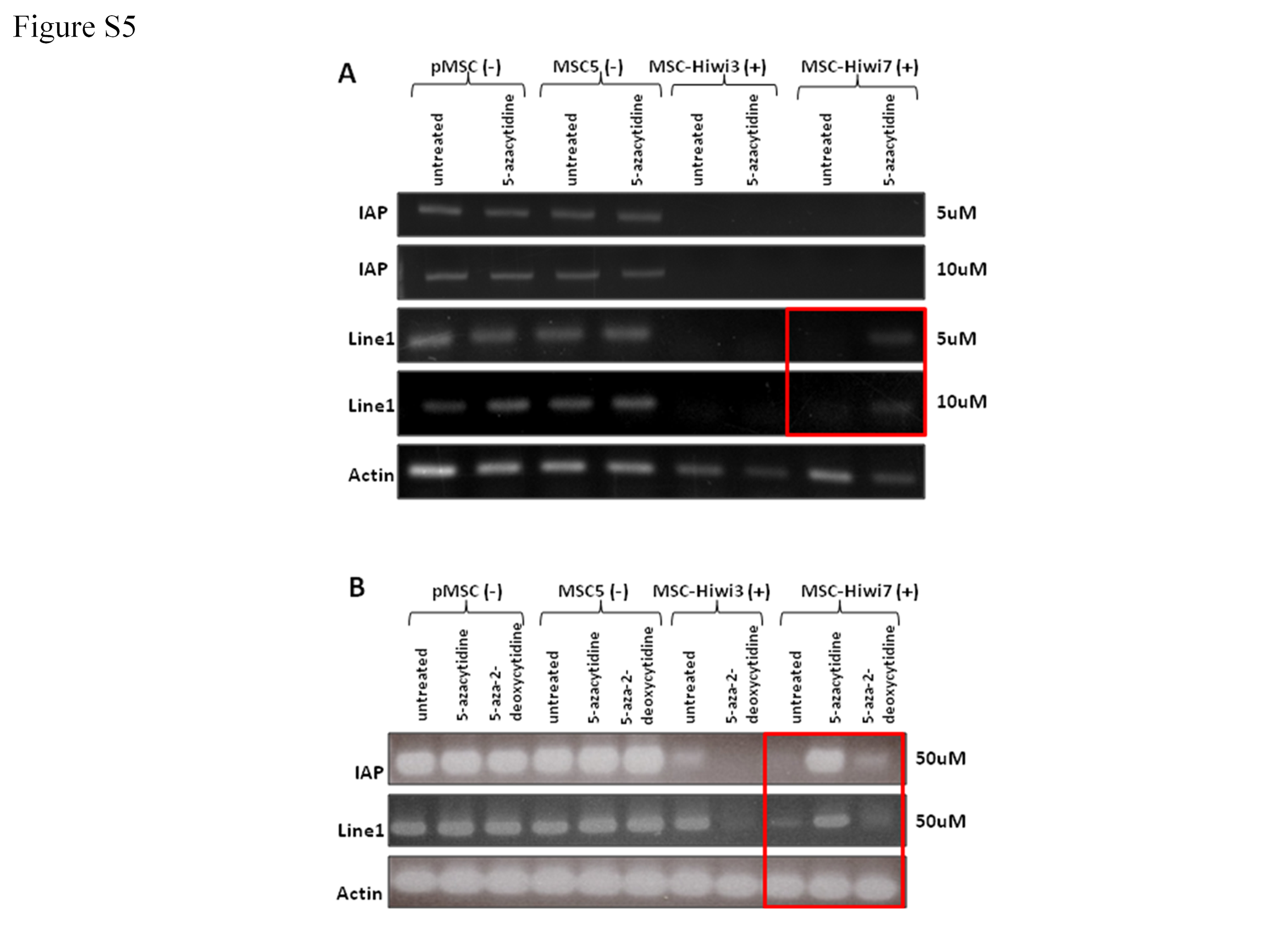

Supplement: Figure S5 — Treatment of Hiwi-MSCs with 5-azacytidine can reverse Hiwi-mediated transposon silencing. (A) Semi-quantitative RT-PCR for Line1 and IAP transposon expression on Hiwi-MSCs treated 18 h with the indicated concentration of 5-azacytidine. Actin is a loading control. Experiments were performed in triplicate. (B) Semi-quantitative RT-PCR for Line1 and IAP transposon expression on Hiwi-MSCs treated 18 h with 50 uM of 5-azacytidine or 5-aza-2-deoxycytidine. Actin is a loading control. Experiments were performed in triplicate. (TIF) [file pone.0033711.s006.tif]

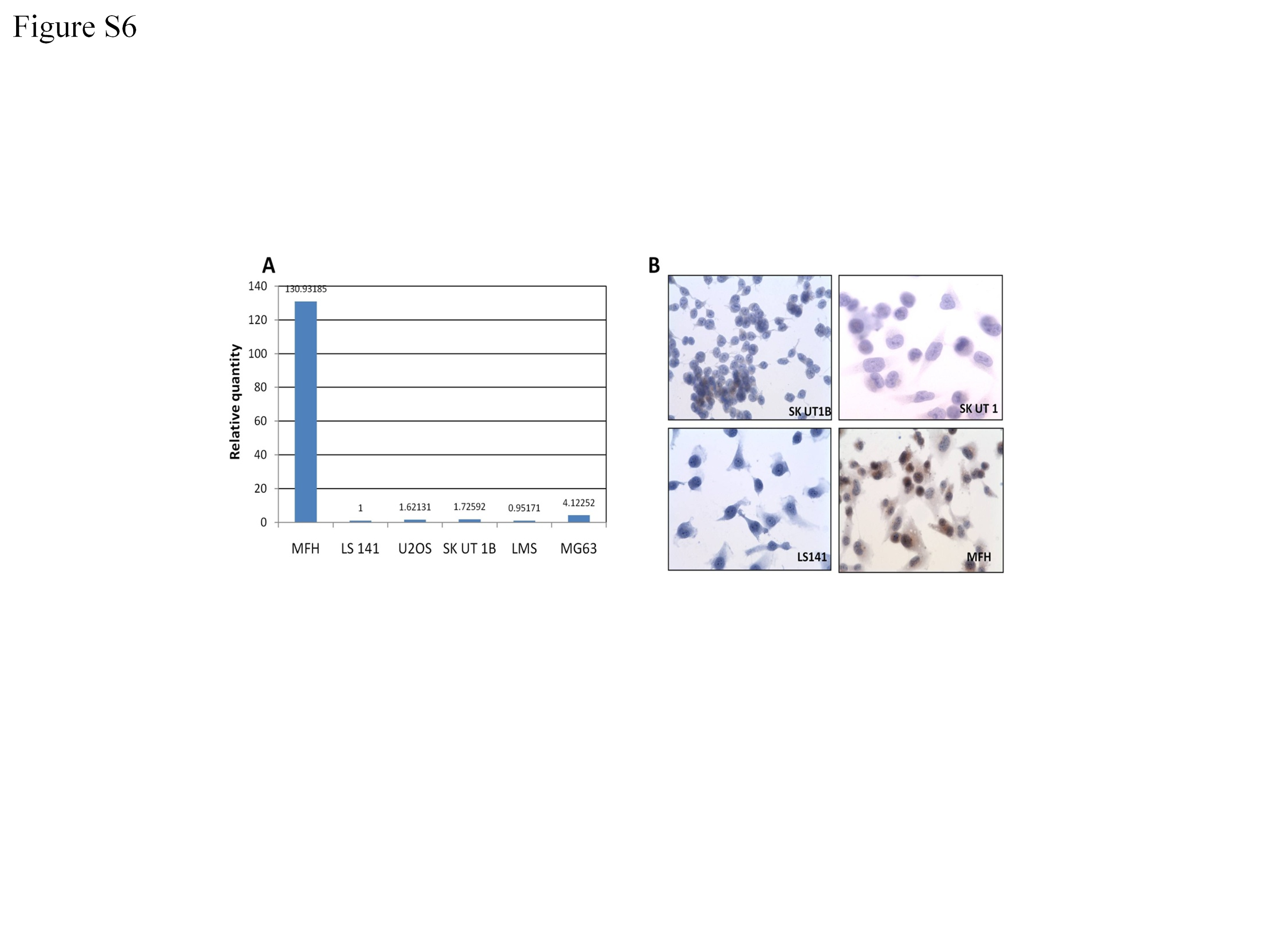

Supplement: Figure S6 — Expression of Hiwi in sarcoma cell lines. (A) Quantitative RT-PCR of Hiwi in a panel of human sarcoma cell lines reveals that MFH has high Hiwi RNA levels. (B) Immunohistochemical analysis of Hiwi in a panel of human sarcoma cell lines reveals that MFH has high Hiwi protein levels. (TIF) [file pone.0033711.s007.tif]

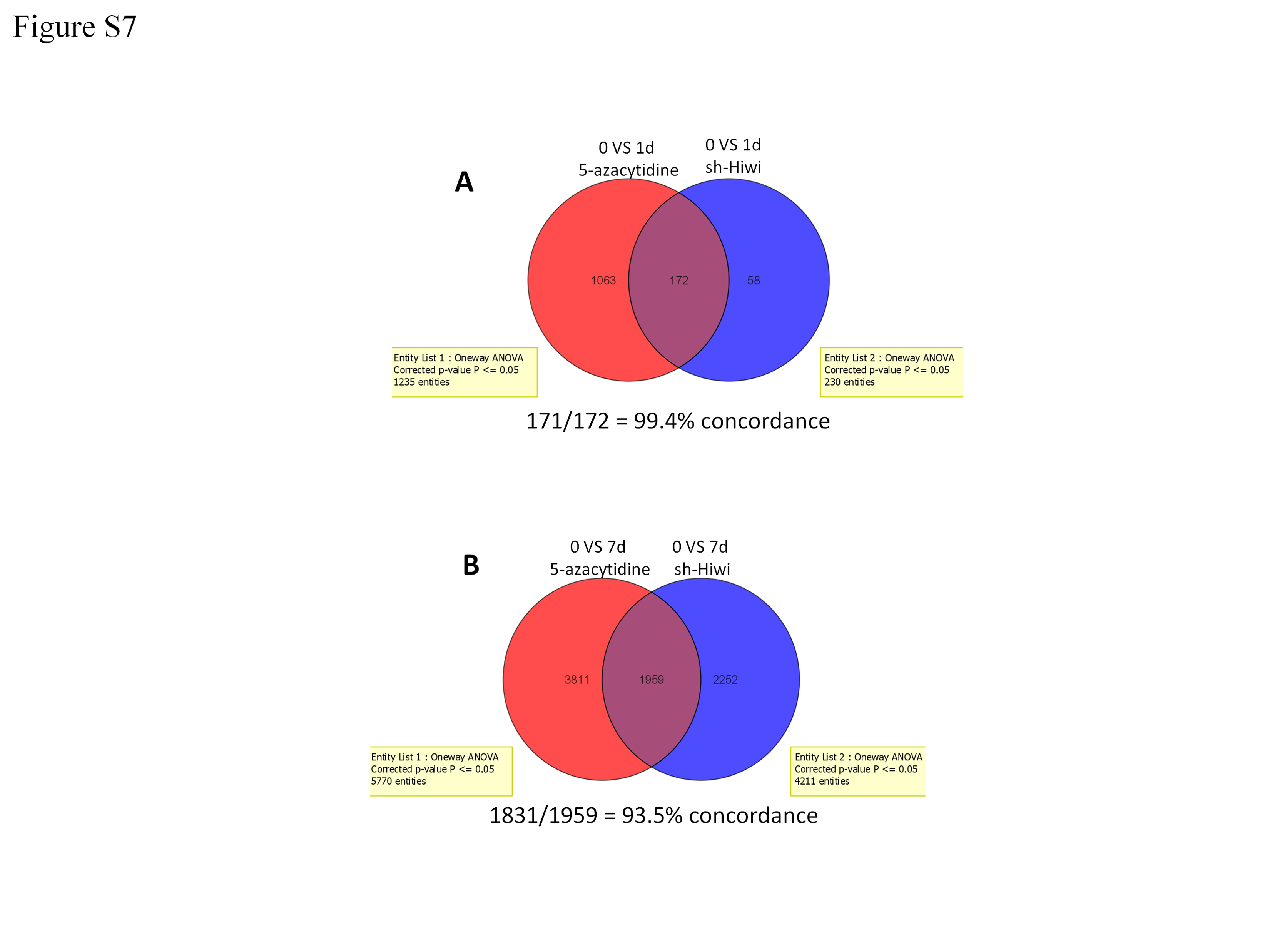

Supplement: Figure S7 — Hiwi down-regulation and 5-azacytidine treatment are mechanistically similar. (A) Venn diagram of overlapping differentially expressed genes in both sh-Hiwi MFH cells and in 5-azacytidine-treated MFH cells. At early time points, over 75% of differentially expressed genes following Hiwi down-regulation are also differentially-expressed during 5-azacytidine treatment. 99% of the overlapping genes move in the same direction in both conditions. (B) At late time points, over 50% of differentially expressed genes following Hiwi down-regulation are also differentially-expressed during 5-azacytidine treatment. 93% of the overlapping genes move in the same direction in both conditions (TIF) [file pone.0033711.s008.tif]

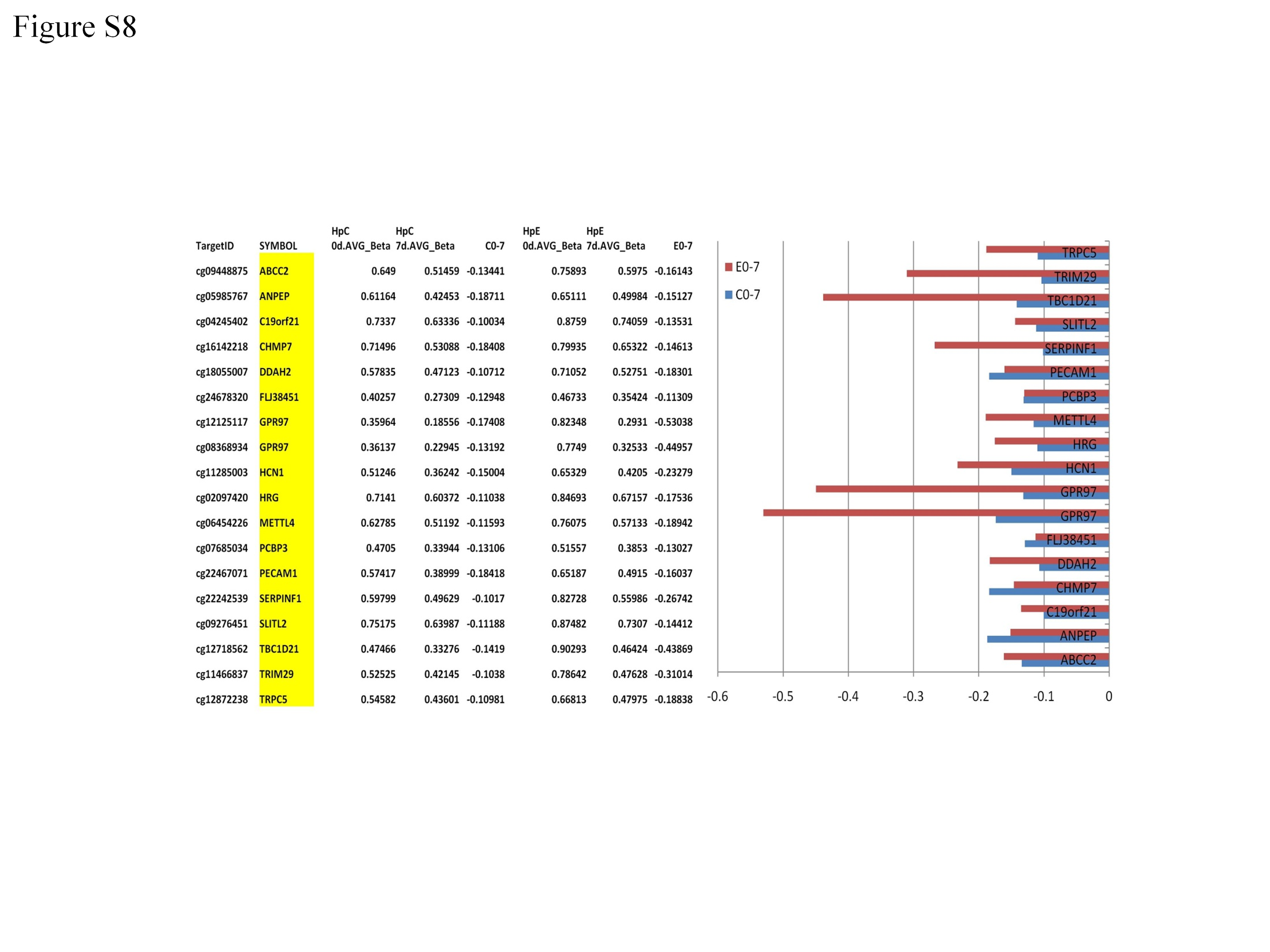

Supplement: Figure S8 — Genes with CpG site promoter hypo-methyation in sh-Hiwi MFH cells. Analysis of Illumina Meth27 promoter methylation arrays reveals only 18 CpG sites that show at least a 10% decrease in methylation after 7 days of doxycycline treatment of sh-Hiwi MFH cells. Genes to which the CpG sites belong are identified here. (TIF) [file pone.0033711.s009.tif]

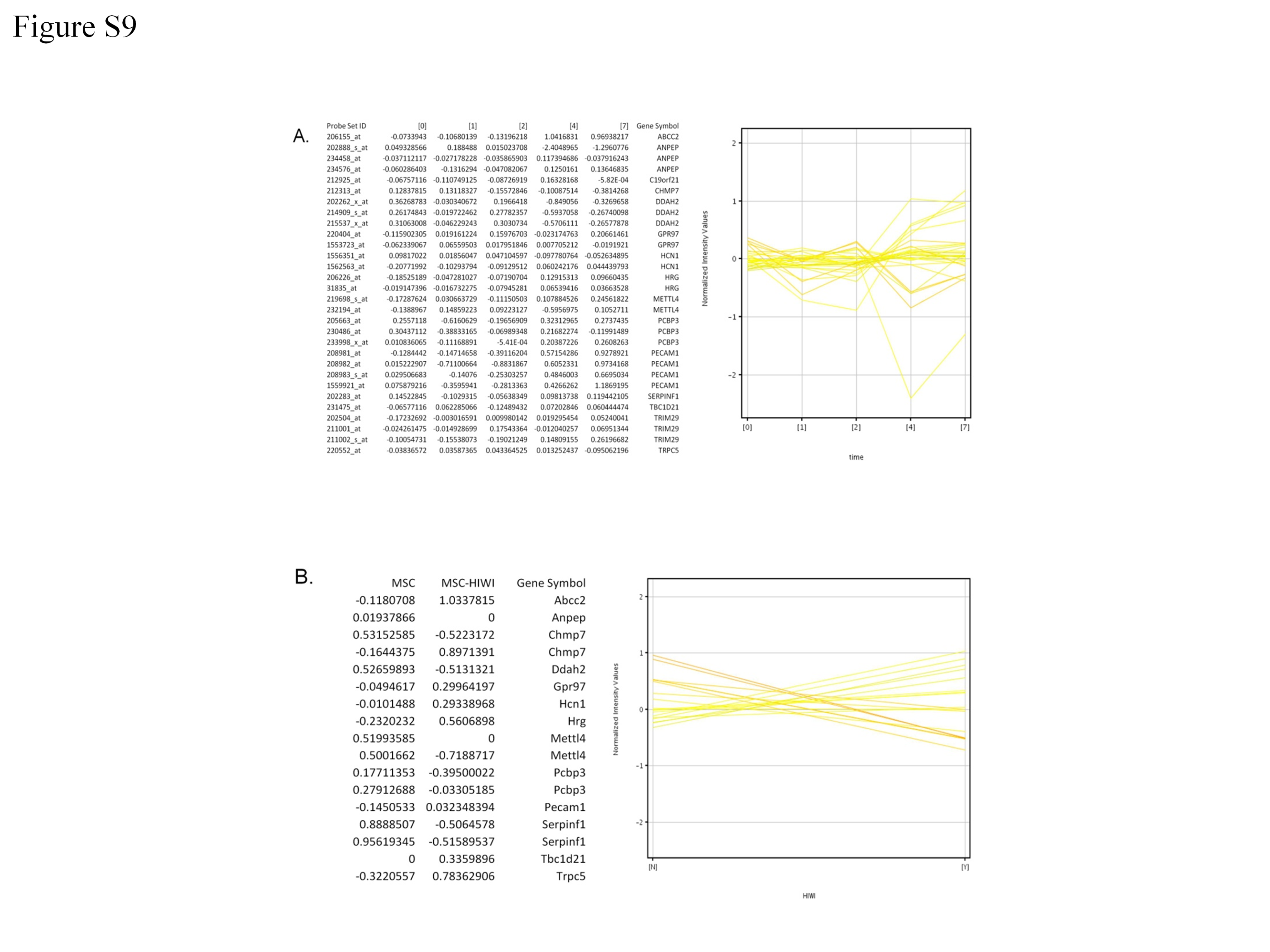

Supplement: Figure S9 — CpG methylated genes do not correlate to genes expression changes in sh-Hiwi MFH cells and Hiwi MSCs. (A) Gene expression profiles of the 17 identified genes with CpG site hypo-methylation, after 0, 2, 4 or 7 days of doxycycline induction of sh-Hiwi MFH cells. While corresponding CpG sites are hypo-methylated, there is no corresponding increase in gene expression. (B) Gene expression profiles of the 17 identified genes with CpG site hypo-methylation in Hiwi-MSCs. Conversely, there is no decrease in expression of these genes. Because each of the 17 identified genes contains multiple spots on the array, corresponding to multiple Gene IDs, multiple rows for each gene are shown in both (A) and (B). (TIF) [file pone.0033711.s010.tif]

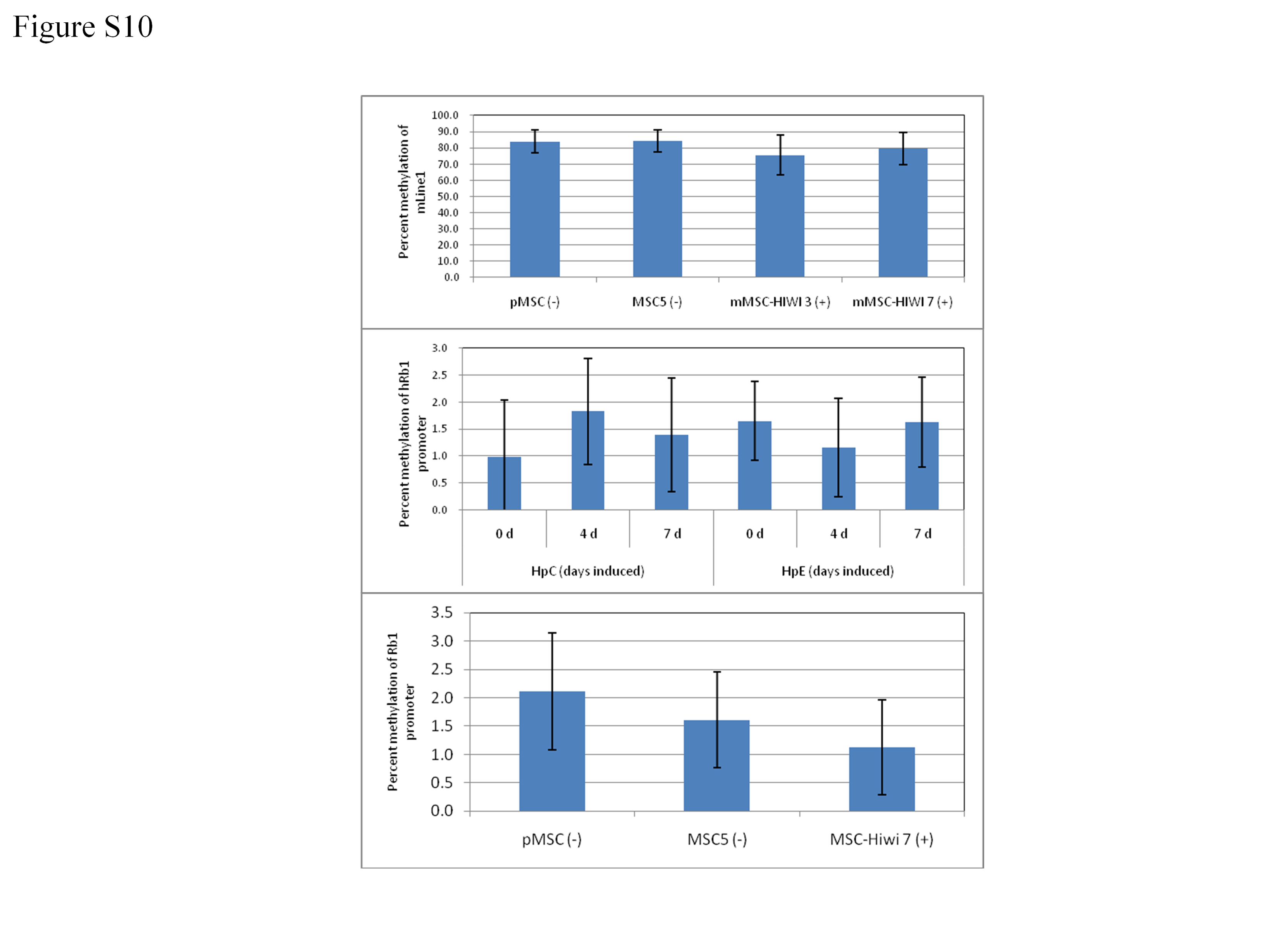

Supplement: Figure S10 — Methylation of Rb1 and Line1 promoter CpG islands do not change as Hiwi levels change. Bisulfite sequencing of Rb1 promoter CpGs in Hiwi-MSCs (top graph) and in sh-Hiwi MFH cells (middle graph) reveal no methylation changes as Hiwi levels change. Similarly, bisulfite sequencing of Line1 CpGs in Hiwi-MSCs (bottom graph) reveal no methylation changes as Hiwi levels change. (TIF) [file pone.0033711.s011.tif]

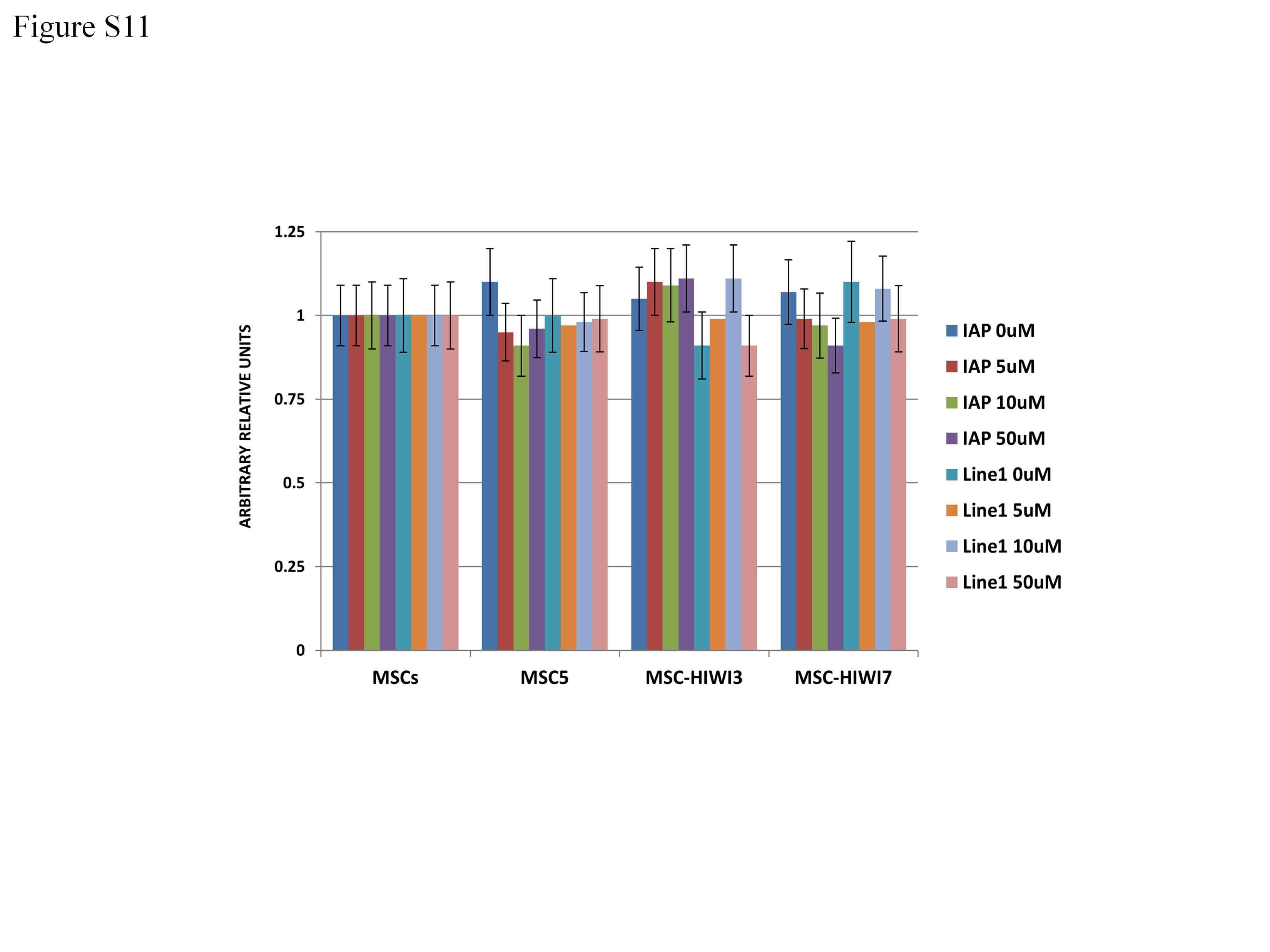

Supplement: Figure S11 — IAP and Line1 transposon methylation is unchanged in Hiwi-MSCs. Quantitative PCR of IAP or Line1 transposon expression, followed by bisulfite conversion, on parental MSCs, MSC5, Hiwi-MSC3 and Hiwi-MSC7 treated with the indicated concentration of 5-azacytidine for 18 h. Experiments were performed in triplicate. Error bars represent standard error. (TIF) [file pone.0033711.s012.tif]

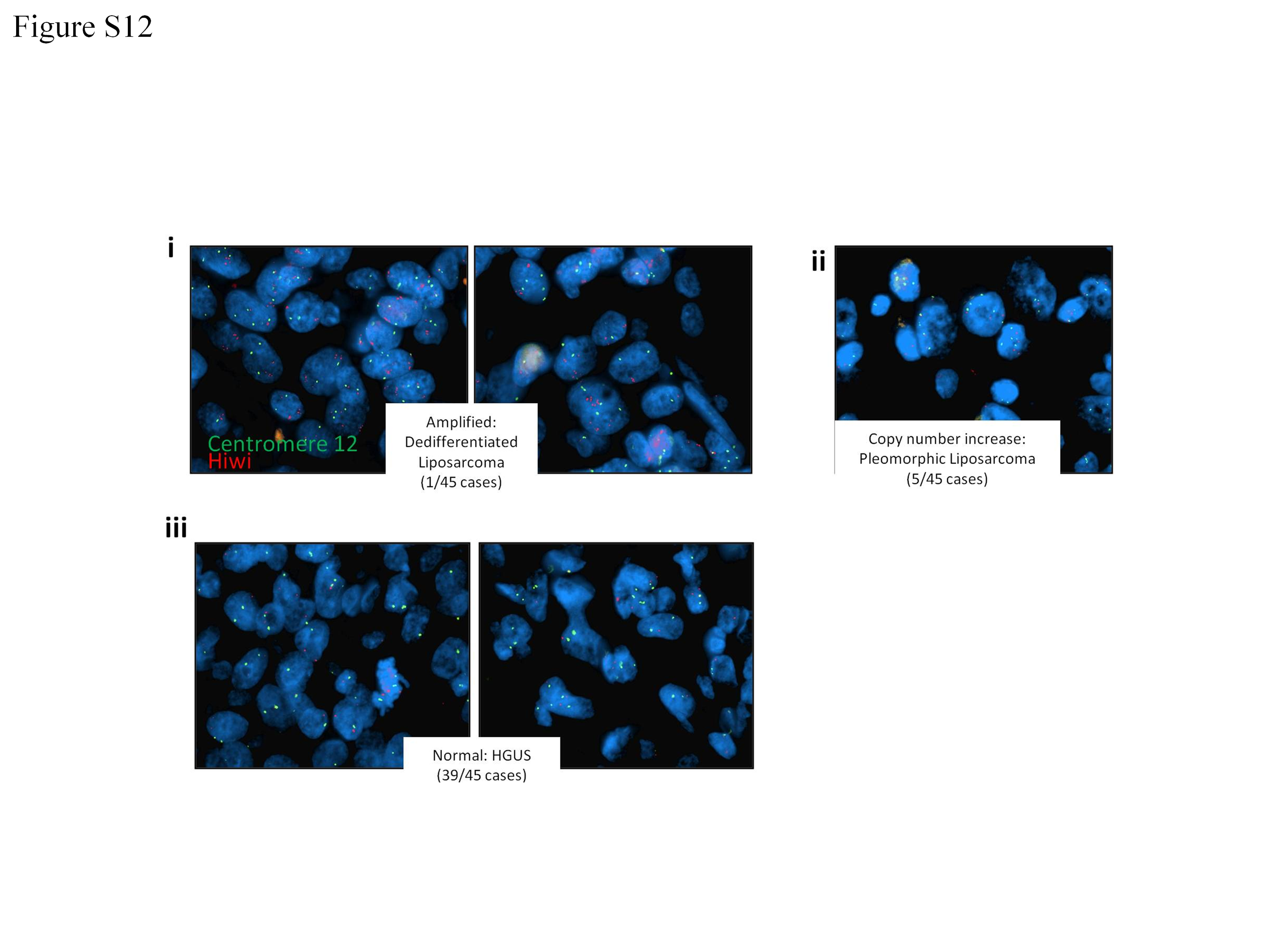

Supplement: Figure S12 — Hiwi is not chromosomally amplified in Hiwi-expressing sarcomas. DNA FISH was performed on the human sarcoma tissue microarray, using a probe against the Hiwi locus on chromosome 12. (i) Analysis of the sarcomas reveals only 1 out of 45 cases that has a true amplification of Hiwi, a dedifferentiated liposarcoma. (ii) A few cases (5 out of 45) have a copy number increase of Hiwi. (iii) However, the majority of cases (39 out of 45), including all HGUS Hiwi-expressing cases, have no chromosomal amplification of Hiwi. (TIF) [file pone.0033711.s013.tif]

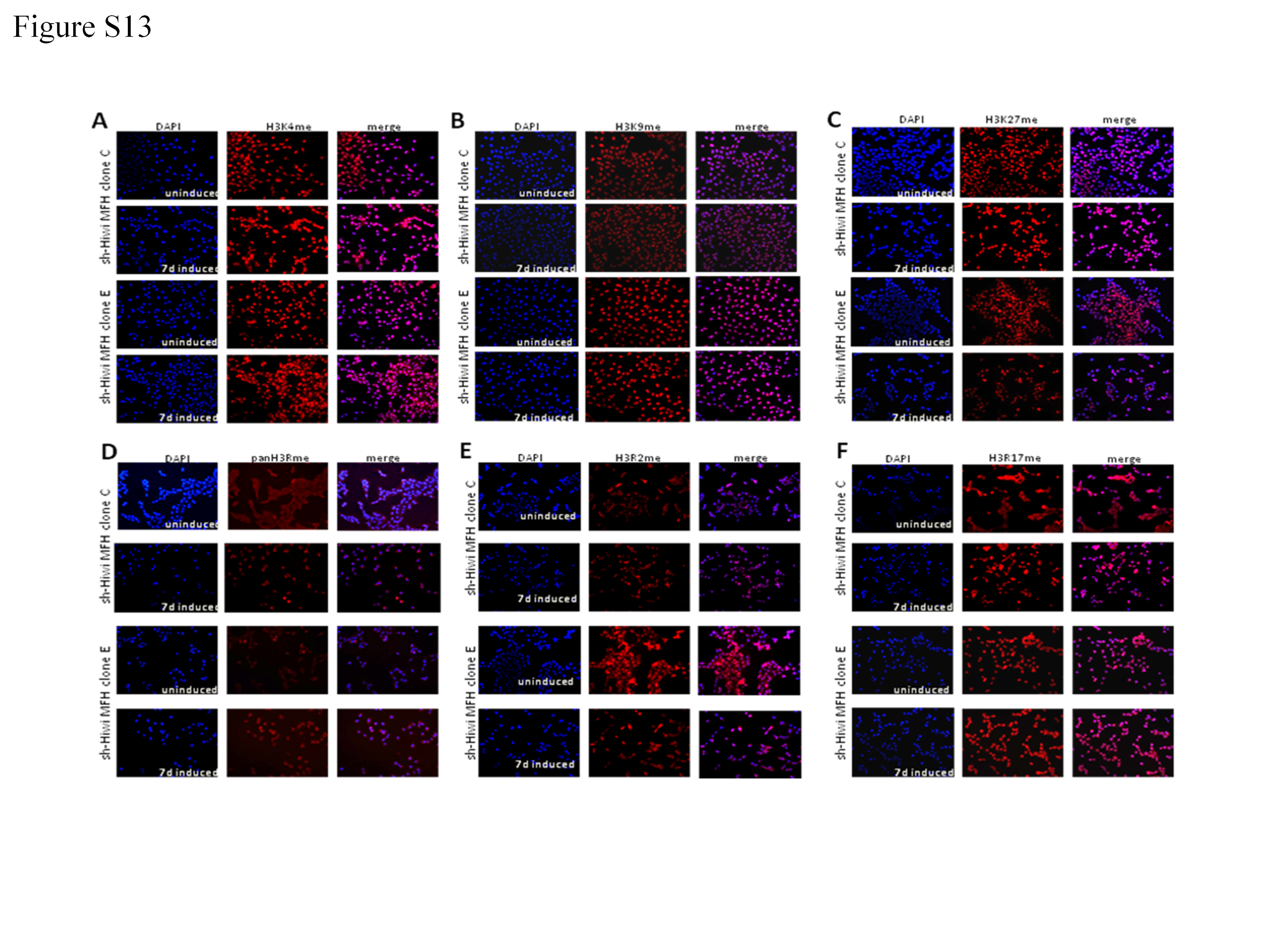

Supplement: Figure S13 — Epigenetic histone marks are unchanged in doxycycline-induced sh-Hiwi MFH cells. Immunofluorescence on sh-Hiwi MFH cells, either uninduced or induced for 7days with doxycycline to knock down Hiwi levels, for the following histone 3 lysine or arginine marks (A) H3K4me; (B) H3K4me; (C) H3K27me; (D) panH3Rme2; (E) H3R2me; (F)H3R17me. (TIF) [file pone.0033711.s014.tif]
